# Supplementary material for: Interventions to reduce social isolation and loneliness during COVID-19 physical distancing measures: A rapid systematic review
Source: PLoS One. 2021 Feb 17;16(2):e0247139. doi: 10.1371/journal.pone.0247139 (PMC7888614; doi:10.1371/journal.pone.0247139)
Supplement: S1 File — (DOCX) [file pone.0247139.s001.docx]

**S1 Appendix:** Full search strategy

The databases Medline (via Ovid), Embase (via Ovid), Cochrane Library, Web of Science (Core Collection) and PsycInfo (via Ebscohost) were searched from inception to April 2020 using the following search terms. Databases were searched in the title, abstract, and keyword fields, and in the MeSH fields when appropriate MeSH terms existed.

(“social isolat*” OR “patient isolat*” OR “emotional isolat*” OR quarantine OR “social distanc*” OR “social support” OR lonel* OR aloneness OR solitude)

AND

(effect* OR efficien* OR evidence OR consequence* OR impact* OR harm* OR outcome*)

AND

(intervention* OR promotion* OR program* OR programme* OR campaign* OR prevention)

AND

(“systematic review*” OR “meta-analys*”)

Results in all databases were limited to English language only.

In addition, the preprint archive MedRxiv was searched using the following search terms, to identify any grey literature relevant to the review subject:

(covid-19 or covid19 or coronavirus or corona virus) and (loneliness or coping or mental health) and (isolation)

The titles and abstracts of articles filed in the MedRxiv COVID-19 and psychiatry and clinical psychology subject were also screened for relevance and those which met inclusion criteria were included.

Full search strategies are reproduced below.

**Medline via Ovid**

(“social isolat*” OR “patient isolat*” OR “emotional isolat*” OR quarantine OR “social distanc*” OR “social support” OR lonel* OR aloneness OR solitude).ti,ab,kw. OR social distance/ or social isolation/ OR patient isolation/ OR quarantine/ OR social support/ OR loneliness/

AND

(effect* OR efficien* OR evidence OR consequence* OR impact* OR harm* OR outcome*).ti,ab,kw.

AND

(intervention* OR promotion* OR program* OR programme* OR campaign* OR prevention).ti,ab,kw. OR health promotion/

AND

(“systematic review*” OR “meta-analys*”).ti,ab,kw. OR systematic review/ OR meta-analysis/

**Embase via Ovid**

(“social isolat*” OR “patient isolat*” OR “emotional isolat*” OR quarantine OR “social distanc*” OR “social support” OR lonel* OR aloneness OR solitude).ti,ab,kw. OR social distance/ or social isolation/ OR patient isolation/ OR quarantine/ OR social support/ OR loneliness/

AND

(effect* OR efficien* OR evidence OR consequence* OR impact* OR harm* OR outcome*).ti,ab,kw.

AND

(intervention* OR promotion* OR program* OR programme* OR campaign* OR prevention).ti,ab,kw. OR health promotion/ OR public health campaign/

AND

(“systematic review*” OR “meta-analys*”).ti,ab,kw. OR systematic review/ OR meta-analysis/

**Web of Science (core collection)**

TS=(“social isolat*” OR “patient isolat*” OR “emotional isolat*” OR quarantine OR “social distanc*” OR “social support” OR lonel* OR aloneness OR solitude)

AND

TS=( effect* OR efficien* OR evidence OR consequence* OR impact* OR harm* OR outcome*)

AND

TS=(intervention* OR promotion* OR program* OR programme* OR campaign* OR prevention)

AND

TS=(“systematic review*” OR “meta-analys*”)

**Cochrane Library**

(“social isolat*” OR “patient isolat*” OR “emotional isolat*” OR quarantine OR “social distanc*” OR “social support” OR lonel* OR aloneness OR solitude):ti,ab,kw OR MeSH Descriptor: [Social Isolation] this term only OR MeSH Descriptor: [Patient Isolation] this term only OR MeSH Descriptor: [Social Distance] this term only OR MeSH Descriptor: [Quarantine] this term only OR MeSH Descriptor: [Social Support] this term only OR MeSH Descriptor: [Loneliness] this term only

AND

(effect* OR efficien* OR evidence OR consequence* OR impact* OR harm* OR outcome*):ti,ab,kw

AND

(intervention* OR promotion* OR program* OR programme* OR campaign* OR prevention):ti,ab,kw OR MeSH Descriptor: [Health Promotion] this term only

AND

(“systematic review*” OR “meta-analys*”):ti,ab,kw OR MeSH Descriptor: [Systematic Review] this term only OR MeSH Descriptor: [Meta-Analysis] this term only

**PsycInfo via Ebscohost**

TI (“social isolat*” OR “patient isolat*” OR “emotional isolat*” OR quarantine OR “social distanc*” OR “social support” OR lonel* OR aloneness OR solitude) OR AB (“social isolat*” OR “patient isolat*” OR “emotional isolat*” OR quarantine OR “social distanc*” OR “social support” OR lonel* OR aloneness OR solitude) OR DE "social isolation" OR DE "social support" OR DE "Loneliness" OR

AND

TI (effect* OR efficien* OR evidence OR consequence* OR impact* OR harm* OR outcome*) OR AB (effect* OR efficien* OR evidence OR consequence* OR impact* OR harm* OR outcome*)

AND

TI (intervention* OR promotion* OR program* OR programme* OR campaign* OR prevention) OR AB (intervention* OR promotion* OR program* OR programme* OR campaign* OR prevention) OR DE "intervention" OR DE "health promotion" OR DE "prevention"

AND

TI (“systematic review*” OR “meta-analys*”) OR AB (“systematic review*” OR “meta-analys*) OR DE "Systematic Review" OR DE "Meta Analysis"

***All databases searched from inception to April 2020, and all results limited to English language only.***

**The preprint archive MedRxiv was also searched for grey literature relating to isolation, mental health, and COVID-19 using the following search string:**

(covid-19 or covid19 or coronavirus or corona virus) and (loneliness or coping or mental health) and (isolation)

| Database name | Number of results |
| --- | --- |
| Medline (via Ovid) | 1285 |
| Embase (via Ovid) | 1830 |
| Web of Science (Core Collection) | 1036 |
| PsycInfo (via Ebscohost) | 560 |
| Cochrane Library | 272 |
| MedRxiv | 2 |

**S1 Table:** Summary characteristics and findings from 23 studies deemed non-feasible with COVID-19 shielding/social distancing guidelines

| ***Author, year***  ***Country*** | ***Target participants & setting*** | ***Age in years:***  ***Mean (range)*** | ***Gender (% female)*** | ***Intervention/Control description*** | ***Duration*** | ***Feasible?*** | ***Loneliness measure*** | ***Loneliness result*** | ***Social isolation/ network measure*** | ***Social isolation/ network result*** | ***Social support measure*** | ***Social support result*** | ***Downs & Black score*** |
| --- | --- | --- | --- | --- | --- | --- | --- | --- | --- | --- | --- | --- | --- |
| **Animal intervention** | | | | | | | | | | | | | |
| Banks, 2002^1^  USA  (RCT) | Residents in long-term care facilities (n = 45) | NREP | 80% | I: Animal assisted therapy – a dog was brought into the long-term care facility, allowing participants to hold, stroke, groom, walk and play with it; visits were either once weekly (I1) or three times weekly (I2)  C: Usual care | 6 w | No – physical contact required | UCLAv3 | 6 w: Significant improvement, η^2^ = 0.206^†^, p = 0.001 | n/a | n/a | n/a | n/a | 16 (Fair) |
| Banks, 2005^2^  USA  (RCT) | Residents from long-term care facilities (n = 33) | 80 (75-90) | 58% | I: Animal assisted therapy – bringing a certified therapy dog into the long-term care facility. The dog always remained on a leash, and each resident was allowed to interact with – talk to, groom, pat – the dog whenever they wanted  C: Group-based AAT with living dog | 6 w | No – physical contact required | UCLAv3 | 5 w: No significant difference between groups, significant improvement in both groups, p < 0.001 | n/a | n/a | n/a | n/a | 17 (Fair) |
| Riddick, 1985^3^  USA  (NRCT) | Non-institutionalised adults ≥55 years in an apartment complex (n = 24) | NREP (57-94) | 77% | I1: Installation of 2.5 gallon goldfish tank; subjects chose two fish for their tank and were visited biweekly. During these visits, researchers spent 15-20 minutes cleaning or assisting with tank maintenance duties and the remaining 10-15 minutes watching and talking about the fish I2: Visitor group  C: Usual care | 6 m | No – physical contact required | UCLA | 6 m: No significant difference, p = 0.08 (ES = -0.25) | n/a | n/a | n/a | n/a | 16 (Fair) |
| Sollami, 2017^4^  Italy  (RCT) | Residents ≥60 years in nursing homes (n = 28) | I: 85.07, C: 84.91 (63-96) | NREP | I: Animal assisted therapy – a dog was introduced to participants in sessions; caregiving activities were gradually performed which included the provision of basic needs, being taught how to physically interact with the dog, and playing fun activities with the dog  C: Usual care | 8 w | No – physical contact required | UCLA | 8 w: Significant improvement, p = 0.000 (ES = 1.45) | n/a | n/a | n/a | n/a | 20 (Good) |
| **Befriending intervention** | | | | | | | | | | | | | |
| MacIntyre, 1999^5^  Canada  (RCT) | New referrals to a community nursing agency's friendly visiting programme (n = 22) | 79.4 (NREP) | 68% | I: Home visit – Volunteer Friendly Visitor Programme – weekly home visits for talking, assisting with care activities, reading, writing letters, and listening  C: Usual care | 6 w | Part feasible – physical contact required | n/a | n/a | n/a | n/a | PRQ | 6 w: PRQ Social integration, Worth subscales – significant improvement (p = 0.03); all other subscales – no significant difference | 19 (Fair) |
| Mulligan, 1978^6^  USA  (NRCT) | Adults ≥65 years (n = 22) | 77 (NREP) | 91% | I: Home visit – Friendly Visitor Program – visits every 2 weeks for 6 months by trained volunteer visitors  C: Usual care | 6 m | No – physical contact required | n/a | n/a | AII/PMI | 6 m: No significant difference (ES = -0.04). 12 m: Significant improvement, p < 0.05 (ES = 0.76) | n/a | n/a | 15 (Fair) |
| Rook, 2003  USA^7^  (NRCT) | Adults ≥60 years (n = 180) | 70.52 (60-92) | 66% | I: Participants were assigned a primary “client” (a child in residence at the state hospital) and given duties typical of a foster grandparent (spending time with them, going on excursions)  C1: Alternative group program; C2: Usual care | 3 y | No – physical contact required | UCLA | 12 m/24 m: No significant difference (1 y ES = 0.06; 2 y ES = 0.11) | # of new relationships formed | 12 m: Significant improvement, ES = 1.42 | n/a | n/a | 19 (Fair) |
| **Educational programme** | | | | | | | | | | | | | |
| Alaviani, 2015^8^  Iran  (RCT) | Older woman 60-74 years old with a moderate loneliness level (n = 150) | NREP | 100% | I: Group sessions where 1) the definitions, causes and complications of loneliness were discussed, 2) Effective Interpersonal interaction was explained, 3) participants were asked to perform social interaction activities such as group walking, and 4) participants visited an elderly nursing home and a Children Charity Organisation  C: Usual care | 2 w | Part feasible – sessions 1 and 2 possible using audio/video call software; session 3 and 4 are not due to requirement for physical contact | UCLA | 6 w: Significant improvement, p < 0.001 (ES = 3.43) | n/a | n/a | n/a | n/a | 19 (Fair) |
| Honigh – de Vlaming, 2013^9^  Netherlands  (NRCT) | Non-institutionalised adults ≥65 years (n = 1804) | I: 73.6, C: 73.8 (NREP) | I: 56%, C: 53% | I: Healthy ageing – five intervention components – a mass media campaign, information meetings, psychosocial group courses, social activation and training of intermediaries  C: Control community | 2 y | Part feasible – multi-component intervention program | DJGL | 24 m: No significant difference, p = 0.67 (ES = 0.10) | n/a | n/a | SSL-I | 24 m: No significant difference, p = 0.59 (ES = 0.05) | 22 (Good) |
| Lupton, 2005^10^  Norway  (NRCT) | Fishing community (n = 1638) | Males:  I = 47.7, C = 48.6 Females: I = 47.5, C = 47.9  (20-62) | 49% | I: Health and Well-Being – population level health intervention  C: Control community | 3 y | Part feasible – multi-component intervention program | % Lonely | 6 y: High risk males (p = 0.004) and low risk females (p = 0.019) - significantly improved, low risk males - no significant difference (p = 0.963) | n/a | n/a | n/a | n/a | 14 (Poor) |
| **Health and Social Care provision** | | | | | | | | | | | | | |
| de Craen, 2006^11^  Netherlands  (RCT) | 85 year old adults (n = 402) | 85 only | I: 64%, C: 67% | I: Home visit – Occupational therapist visit providing training and education about assistive devices that were already present, as well as information about assistive devices that might benefit them  C: Usual care | Single visit | No – physical contact required | DJGL | 6 m/24 m: No significant difference (p = 0.78, p = 0.92) | n/a | n/a | n/a | n/a | 24 (Good) |
| Melis, 2008^12^  Netherlands  (RCT) | Adults ≥70 years living in own home or retirement home (n = 151) | I: 81.7, C: 82.8 (NREP) | 75% | I: Home visit – Dutch Geriatric Intervention Program (DGIP) – geriatric specialist nurse visit to develop an individualised, integrated treatment plan for each patient  C: Usual care | 3 m | No – physical contact required | DJGL | 3 m: No significant difference, p > 0.05 | n/a | n/a | n/a | n/a | 20 (Good) |
| Clarke, 1992^13^  UK  (RCT) | Elderly people ≥75 living alone (n = 523) | NREP | NREP | I: Personalised assistance/support – the type of assistance given varied but was tailored to each person's request for help. Types of assistance includes social services, financial (collecting benefits or pensions), housing (installation of safety chains onto doors, decorating), nursing and medical  C: Usual care | 1.25 to 2 y | Part feasible – multi-component intervention program | WLS | 3 y: No significant difference | n/a | n/a | n/a | n/a | 22 (Good) |
| McEwan, 1990^14^  UK  (RCT) | Elderly people ≥75 years (n = 296) | NREP | NREP | I: Screening program – Home visit from a nurse at which an assessment was carried out on activities of daily living, social functioning, sensory functions, mental and emotional problems, current medical problems, blood pressure, urinalysis, haemoglobin level and compliance with medication  C: Usual care | Single visit | No – physical contact required | n/a | n/a | NHP-I | 20 m: Significant improvement, p < 0.01 | n/a | n/a | 18 (Fair) |
| Morrow-Howell, 1998^15^  USA  (RCT) | Adults ≥60 years (n = 80) | 76 (61-92) | 85% | I: Telephone-mediated Link-Plus program consisting of a multidimensional assessment of physical, mental and social health; service arrangement in response to identified needs; ongoing telephone contact and supportive therapy encouraging the client to articulate and solve problems, and identify strengths and successful coping mechanisms  C: Usual care | 4 m | Part feasible – services arranged involved housekeepers coming to the client's home, provision of transport to doctor's appointments | Frequency of loneliness | 4 m: No significant difference, p = 0.36 | n/a | n/a | n/a | n/a | 19 (Fair) |
| Ollonqvist, 2008^16^  Finland  (RCT) | Adults ≥65 years living at home but with risk of institutionalisation within 2 years (n = 708) | I: 78.1, C: 78.6 (65-96) | 86% | I: Geriatric rehabilitation program – group activities focused on physical activation such as exercises in a sitting position, pool exercises or resistance training in the gym. Participants also attended group discussions and lectures on older people's life situation and possible problems, promotion of self-care, psychological counselling and information on social services and recreational activities  C: Usual care | 8 m | No – physical contact required | “Do you feel lonely?” | 12 m: Non-significant improvement | n/a | n/a | n/a | n/a | 22 (Good) |
| Parsons, 2013^17^  New Zealand  (RCT) | Community-dwelling adults ≥65 years (≥55 years if Maori or Pacific Islander) (n = 205) | I: 79.1, C: 76.90 (NREP) | I: 71%, C: 61% | I: Home care – use of goal-setting tool to identify participant goals and use these to structure home care support services  C: Usual care | 6 m | No – physical contact required | n/a | n/a | n/a | n/a | DSSI | 6 m: No significant difference, p = 0.09 (ES = 0.90) | 25 (Good) |
| **Leisure/skill development intervention** | | | | | | | | | | | | | |
| Tse, 2014^18^  Hong Kong  (RCT) | Older residents of nursing homes with chronic pain (n = 396) | I: 85.45, C: 85.44 (NREP) | I: 81%, C: 79% | I: Exercise Program – weekly 1 hour classes with different types of exercise offered each session  C: Usual care | 8 w | No – physical contact required | UCLAv3 | 8 w: Significant improvement, p = 0.000 (ES = 0.15) | n/a | n/a | n/a | n/a | 19 (Fair) |
| Cohen, 2006^19^  USA  (NRCT) | Adults ≥64 years (n = 166) | I: 79.0, C: 79.6 (NREP) | I: 78%, C: 80% | I: Professionally conducted chorale with weekly singing rehearsals and public performances  C: Usual care | 30 w | No – physical contact required | UCLA | 12 m: No significant difference, p = 0.08 (ES = -0.08) | n/a | n/a | n/a | n/a | 16 (Fair) |
| Yap, 2017^20^  Singapore  (RCT) | Adults ≥65 years (n = 51) | 74.65 (NREP) | 94% | I: Rhythm-centred music making (RMM) – participants seated in a circle use drums or percussion instruments to play freely or interact with each other through the active playing of the instruments  C: Usual care | 11 w | No – physical contact required | n/a | n/a | LSNS | 11 w: No significant difference, p = 0.329 | n/a | n/a | 23 (Good) |
| Pynnonen, 2018^21^  Finland  (RCT) | Adults 75-79 years old living in the city centre (n = 257) | 77.0 (NREP) | 75% | I: Intervention group allowed to select 3 activities: exercise program, personal counselling, and social activity program. Exercise program involved varying types of exercise in a gym. Social activity program activities included group discussions, self-expression using art, and going on day-trips. Personal counselling was conducted by a rehabilitation counsellor  C: Usual care | 6 m | No – physical contact required | % Lonely | 3 m/6 m/12 m: No significant difference, p = 0.896 | n/a | n/a | SPS-SI | 3 m/6 m/12 m: Significant improvement | 21 (Good) |
| Routasalo, 2009^22^  Finland  (RCT) | Adults ≥75 years suffering from loneliness (n = 235) | I: 80, C: 80 (75-92) | I: 74%, C: 72% | I: Weekly psychosocial group intervention consisting of three types of activities depending on participant interest: art and inspiring activities (AIA), group exercise and discussions (GED), and therapeutic writing and group therapy (TWGT)  C: Usual care | 3 m | Part feasible – TWGT (feasible) using audio/video call software; AIA (not feasible) involves artist visits/visiting cultural events; GED (not feasible) involves group exercise | UCLAv3 | 3 m/6 m: No significant difference | LSNS | No significant difference, p = 0.50 (which f/u not specified) | n/a | n/a | 19 (Fair) |
| **Social facilitation** | | | | | | | | | | | | | |
| Boen, 2012^23^  Norway  (RCT) | Adults ≥65 years living at home (n = 138) | NREP | I: 56%, C: 55% | I: Senior centre group programme – self-help group discussing topics such as safety in the home, social relations, humour and laughter + physical training programme  C: Usual care | 12 m | Part feasible – multi-component intervention program | n/a | n/a | n/a | n/a | OSSS-3 | 12 m: No significant difference, p < 0.08, Cohen's d = 0.12^†^ (ES = 0.08) | 18 (Fair) |

AII/PMI=Adulthood Isolation Index/Past Month Isolation Index; DJGL=de Jong Gierveld loneliness scale; DSSI=Duke Social Support Index; ES=Effect size (standardised mean difference); LSNS=Lubben Social Network Scale; NHP-I=Isolation subscale of Nottingham Health Profile; NRCT=Non-randomised controlled trial; NREP=not reported; OSSS-3=Oslo-3 Social Support scale; PRQ=Personal Resource Questionnaire; RCT=Randomised controlled trial; SPS-SI=Social integration subscale of Social Provisions Scale; SSL-I=Social Support List-Interactions; UCLA=University California Los Angeles loneliness scale (1980); UCLAv3=University California Los Angeles loneliness scale version 3 (1996); WLS=Wenger's Loneliness scale. d/w/m/y=day/week/month/year; I=intervention, C=control; n/a=not applicable. †Effect size reported in study results.

**S2 Table:** Summary characteristics of the 58 studies deemed feasible with COVID-19 shielding/social distancing guidelines

| ***Author, year*** | ***Country*** | ***Study design*** | ***Target participants & setting*** | ***Age in years***  ***Mean (range)*** | ***Gender (% female)*** | ***Intervention duration*** | ***Downs & Black score*** |
| --- | --- | --- | --- | --- | --- | --- | --- |
| **Animal intervention** | | | | | | | |
| Banks, 2008^24^ | USA | RCT | Elderly residents in long-term care facilities (n = 38) | NREP | NREP | 8 w | 14 (Poor) |
| Jessen, 1996^25^ | USA | RCT | Adults ≥65 years in skilled rehabilitation unit (n = 40) | 76 (65-91) | 68% | 10 d | 19 (Fair) |
| Robinson, 2013^26^ | New Zealand | RCT | Residents in residential care facility (n = 40) | NREP (55-100) | 68% | 12 w | 17 (Fair) |
| **Befriending intervention** | | | | | | | |
| Heller, 1991^27^ | USA | RCT | Residents in low-income housing (n = 291) | 74 (median) (NREP) | 100% | 10 w x2 | 17 (Fair) |
| Mountain, 2014^28,29^ | UK | RCT | Adults aged ≥74 years living independently (n = 70) | I: 81.8, C: 80.1 (NREP) | 59% | 18 w | 22 (Good) |
| Schulz, 1976^30^ | USA | RCT | Residents in a retirement home (n = 40) | 81.5 (67-96) | 86% | 2 m | 14 (Poor) |
| **Educational programme** | | | | | | | |
| Bouwman, 2017^31^ | Netherlands | RCT | Adults ≥50 years (n = 239) | 61.58 (50-86) | 78% | 10 w | 18 (Fair) |
| Martina, 2006^32,33^ | Netherlands | NRCT | Women ≥55 years (n = 115) | 63 (53-86) | 100% | 12 w | 15 (Fair) |
| Tilburg, 2000^34^ | Netherlands | NRCT | Participants in the friendship program for older women (n = 64) | 63.2 (54-80) | 100% | NREP | 16 (Fair) |
| Cohen-Mansfield, 2018^35^ | Israel | RCT | Older adults (n = 74) | I: 76.6, C: 79 (66-92) | 81% | 6 m | 19 (Fair) |
| Kremers, 2006^36^ | Netherlands | RCT | Single women ≥55 years (n = 142) | NREP | 100% | 6 w | 19 (Fair) |
| Mountain, 2017^37^ | UK | RCT | Adults ≥65 years (n = 288) | 72.1 (65-92) | 68% | 4 m | 21 (Good) |
| Ruffing-Rahal, 1994^38^ | USA | NRCT | Independent adults ≥65 years in the community (n = 28) | I: 76.93, C: 76.79 (NREP) | 82% | 6 m | 19 (Fair) |
| Seepersad, 2005^39^ | USA | NRCT | College students (n = 380) | 21 (NREP) | 74% | 7 w | 15 (Fair) |
| **Health and Social Care provision** | | | | | | | |
| Hall, 1992^40^ | Canada | RCT | Adults ≥65 years living in their own home (n = 167) | I: 78.0, C: 77.9 (NREP) | I: 79%, C: 78% | 3 y | 21 (Good) |
| van Rossum, 1993^41^ | Netherlands | RCT | Elderly people aged 75-84 years old living at home (n = 580) | NREP (75-84) | I: 58%, C: 57% | 3 y | 17 (Fair) |
| Dickens, 2011^42^ | UK | NRCT | Adults ≥50 years (n = 393) | I: 71.8, C: 69.8 (NREP) | I: 69%, C: 61% | 12 w | 19 (Fair) |
| **Leisure/skill development intervention** | | | | | | | |
| Shapira, 2007^43^ | Israel | NRCT | Older adults in day-care centres or nursing homes (n = 48) | I: 80.25 (70-93), C: 82.60 (70-93) | 63% | 15 w | 17 (Fair) |
| Slegers, 2008^44^ | Netherlands | RCT | Adults 64-75 years old with no prior computer experience (n = 236) | NREP | NREP | 12 m | 16 (Fair) |
| White, 1999^45^ | USA | NRCT | Older adults in a retirement community (n = 27) | I: 77, C: 80 (NREP) | I: 84%, C: 75% | 4 m | 16 (Fair) |
| White, 2002^46^ | USA | RCT | Residents of 4 congregate housing sites and 2 nursing facilities (n = 100) | I: 71, C: 72 (NREP) | I: 71%, C: 82% | 5 m | 17 (Fair) |
| Woodward, 2011^47^ | USA | RCT | Adults ≥60 years (n = 82) | 72 (60-89) | 72% | 6 m | 16 (Fair) |
| Bickmore, 2005^48^ | USA | RCT | Referrals from the Geriatric Ambulatory Practice clinic (n = 21) | 74.0 (63-85) | 86% | 2 m | 17 (Fair) |
| Dowd, 2014^49^ | Canada | RCT | Inactive adults at a large university (n = 84) | 21.96 (NREP) | 73% | 2 w | 18 (Fair) |
| Kamegaya, 2014^50^ | Japan | RCT | Adults ≥65 years living independently (n = 52) | 74.9 (NREP) | 90% | 12 w | 19 (Fair) |
| Iliffe, 2014^51^ | UK | RCT | Adults ≥65 years living independently (n = 953) | 73 (65-94) | 62% | 24 w | 22 (Good) |
| Maki, 2012^52^ | Japan | RCT | Adults ≥65 years (n = 150) | 72.0 (NREP) | 71% | 3 m | 19 (Fair) |
| McAuley, 2000^53^ | USA | RCT | Sedentary adults 60-75 years old (n = 174) | 66.71 (NREP) | 71% | 6 m | 17 (Fair) |
| Mutrie, 2012^54^ | UK | RCT | Adults ≥65 years living independently (n = 41) | I: 71.6, C: 70.0 (65-83) | 68% | 12 w | 20 (Good) |
| Tarazona-Santabalbina, 2016^55^ | Spain | RCT | Community-dwelling, sedentary adults ≥70 years (n = 100) | I: 79.7, C: 80.3 (NREP) | 54% | 24 w | 23 (Good) |
| Perkins, 2012^56^ | USA | NRCT | Community-dwelling older adults (n = 34) | 72 (57-87) | 97% | 6 w | 14 (Poor) |
| Tse, 2010^57^ | Hong Kong | RCT | Residents ≥60 years in nursing homes (n = 53) | I: 85, C: 83 (65-95) | 85% | 8 w | 17 (Fair) |
| Bell, 2011^58^ | USA | NRCT | Residents ≥60 years in assisted living facilities (n = 22) | NREP (60-94) | 73% | 8 w | 17 (Fair) |
| Kahlbaugh, 2011^59^ | USA | RCT | Elderly people in independent living residential apartments (n = 35) | 82 (NREP) | 89% | 10 w | 18 (Fair) |
| Jung, 2009^60^ | Singapore | RCT | Elderly people ≥60 years in long-term care facility (n = 45) | NREP (56-92) | NREP | 6 w | 15 (Fair) |
| Xu, 2016^61^ | Singapore | NRCT | Older adults at Senior Activities Centre (n = 89) | I1 = 75.9, I2 = 76.0, C = 73.1 (NREP) | 77% | 1 w | 15 (Fair) |
| Winstead, 2014^62^ | USA | RCT | Residents living in assisted and independent living facilities (n = 141) | 83.24 (NREP) | 81% | 8 w | 19 (Fair) |
| **Psychological therapy** | | | | | | | |
| Cobb, 2014^63^ | USA | RCT | Adults ≥18 years (n = 1502) | I: 42.4, C: 42.6 (NREP) | 70% | 3 m | 20 (Good) |
| Conoley, 1985^64^ | USA | RCT | Female undergraduate psychology volunteers (n = 57) | NREP | 100% | 1 w | 17 (Fair) |
| Dodge, 2015^65^ | USA | RCT | Adults living in retirement communities or going to Senior centres (n = 83) | 80.5 (NREP) | 76% | 6 w | 22 (Good) |
| McWhirter, 1996^66^ | USA | RCT | Lonely volunteers at a university counselling centre (n = 44) | 24.8 (18-38) | 48% | 6 w | 17 (Fair) |
| Winningham, 2007^67^ | USA | NRCT | Residents in assisted living facility (n = 58) | 82.11 (61-98) | NREP | 3 m | 13 (Poor) |
| Kuru Alici, 2018^68^ | Turkey | NRCT | Adults ≥65 years living in nursing homes (n = 72) | NREP (65-97) | 54% | 5 w | 18 (Fair) |
| Elsherbiny, 2018^69^ | Egypt | RCT | Residents 60-70 years old at an elderly care institution (n = 43) | 68.2 (NREP) | 33% | 12 w | 18 (Fair) |
| Adair, 2018^70^ | USA | RCT | Adults in the UNC and Chapel Hill community (n = 94) | 40.09 (18-65) | 82% | 6 w | 18 (Fair) |
| Creswell, 2012^71^ | USA | RCT | Healthy older adults (n = 40) | 65 (55-85) | 83% | 8 w | 23 (Good) |
| Zhang, 2018^72^ | China | RCT | College students with elevated loneliness (n = 50) | I: 20.38, C: 19.19 (17-25) | I: 47%, C: 31% | 8 w | 20 (Good) |
| Chiang, 2010^73^ | Taiwan | RCT | Adults ≥65 years in a nursing home (n = 110) | 77.24 (NREP) | 0% | 2 m | 19 (Fair) |
| Westerhof, 2018^74^ | Netherlands | RCT | Adults ≥60 years in a care facility (n = 81) | 84.2 (60-98) | 62% | 8 w | 19 (Fair) |
| Chan, 2017^75^ | Hong Kong | RCT | Adults ≥60 years (n = 48) | 77.3 (66-103) | 76% | 3 m | 21 (Good) |
| Wikstrom, 2002^76^ | Sweden | RCT | Female tenants with moderate needs for assistance in specifically designed flats (n = 40) | 82.6 (70-97) | 100% | 4 m | 13 (Poor) |
| **Social facilitation** | | | | | | | |
| Andersson, 1985^77^ | Sweden | RCT | Older women who have requested admission to senior citizen apartments (n = 108) | 77 (NREP) | 100% | 6 m | 13 (Poor) |
| Baumgarten, 1988^78^ | Canada | NRCT | Elderly residents of a government-subsidised apartment building (n = 95) | NREP | I: 75%, C: 71% | 15 m | 16 (Fair) |
| Czaja, 2018^79^ | USA | RCT | Individuals ≥65 years living alone (n = 300) | 76.15 (65-98) | 78% | 12 m | 23 (Good) |
| Lokk, 1990^80^ | Sweden | RCT | Elderly people in a day-care unit (n = 65) | NREP (61-90) | 49% | 12 w | 14 (Poor) |
| Saito, 2012^81^ | Japan | RCT | Adults ≥65 years who experienced relocation within 2 years (n = 60) | I: 72.5, C: 72.8 (66-84) | 67% | 6 w | 22 (Good) |
| Tsai, 2010^82^ | Taiwan | RCT | Residents in nursing homes (n = 57) | I: 74.42, C: 78.48 (NREP) | 58% | 3 m | 18 (Fair) |
| Tsai, 2011^83^ | Taiwan | RCT | Residents in nursing homes (n = 90) | 73.82 (NREP) | 55% | 3 m | 20 (Good) |

RCT=randomised controlled trial; NRCT=non-randomised controlled trial; I=intervention; C=control; NREP=not reported; d/w/m/y = day/week/month/year

**S3 Table:** Risk of bias assessment performed using the Downs and Black tool. All 81 studies, including both feasible and non-feasible interventions, were assessed for risk of bias. All questions were answered following the authors’ guidelines except for question 27, which was adapted to, “Was an attempt made to calculate the power of the study, or the sample size needed for sufficient power?”. Score ranges were given the following quality levels: excellent (26–28); good (20–25); fair (15–19); and poor (≤14).

|  |  | **Reporting** | | | | | | | | | | **External Validity** | | | **Internal validity - bias** | | | | | | | **Internal validity - confounding** | | | | | | **Power** | **Total** |  |
| --- | --- | --- | --- | --- | --- | --- | --- | --- | --- | --- | --- | --- | --- | --- | --- | --- | --- | --- | --- | --- | --- | --- | --- | --- | --- | --- | --- | --- | --- | --- |
| **Author** | **Year** | **1** | **2** | **3** | **4** | **5** | **6** | **7** | **8** | **9** | **10** | **11** | **12** | **13** | **14** | **15** | **16** | **17** | **18** | **19** | **20** | **21** | **22** | **23** | **24** | **25** | **26** | **27** | **(/28)** | **Rating** |
| **Feasible Interventions** | | | | | | | | | | | | | | | | | | | | | | | | | | | | | | |
| Adair^70^ | 2018 | 1 | 1 | 1 | 1 | 2 | 0 | 0 | 0 | 0 | 1 | 0 | 0 | 1 | 1 | 1 | 1 | 1 | 0 | 1 | 1 | 1 | 1 | 1 | 0 | 0 | 0 | 1 | 18 | Fair |
| Andersson^77^ | 1985 | 1 | 1 | 1 | 1 | 0 | 0 | 0 | 0 | 0 | 0 | 0 | 0 | 1 | 0 | 0 | 1 | 1 | 1 | 1 | 0 | 1 | 1 | 1 | 0 | 1 | 0 | 0 | 13 | Poor |
| Banks^24^ | 2008 | 1 | 1 | 1 | 1 | 0 | 0 | 1 | 0 | 0 | 0 | 0 | 0 | 1 | 0 | 0 | 1 | 1 | 1 | 1 | 1 | 1 | 1 | 1 | 0 | 0 | 0 | 0 | 14 | Poor |
| Baumgarten^78^ | 1988 | 1 | 1 | 1 | 1 | 2 | 1 | 0 | 0 | 0 | 1 | 0 | 0 | 1 | 0 | 0 | 1 | 1 | 1 | 1 | 1 | 0 | 1 | 0 | 0 | 1 | 0 | 0 | 16 | Fair |
| Bell^58^ | 2011 | 1 | 1 | 1 | 1 | 1 | 0 | 1 | 0 | 1 | 1 | 0 | 0 | 1 | 0 | 0 | 1 | 1 | 1 | 1 | 1 | 1 | 1 | 0 | 0 | 0 | 1 | 0 | 17 | Fair |
| Bickmore^48^ | 2005 | 1 | 1 | 1 | 1 | 2 | 0 | 0 | 0 | 0 | 1 | 0 | 0 | 1 | 0 | 0 | 1 | 1 | 1 | 1 | 1 | 1 | 1 | 1 | 0 | 0 | 1 | 0 | 17 | Fair |
| Bouwman^31^ | 2017 | 1 | 1 | 1 | 1 | 0 | 1 | 1 | 0 | 1 | 0 | 0 | 0 | 1 | 1 | 0 | 1 | 1 | 1 | 1 | 1 | 1 | 1 | 1 | 0 | 0 | 1 | 0 | 18 | Fair |
| Chan^75^ | 2017 | 1 | 1 | 1 | 1 | 2 | 1 | 1 | 0 | 0 | 1 | 0 | 0 | 1 | 0 | 1 | 1 | 1 | 1 | 1 | 1 | 1 | 1 | 1 | 1 | 1 | 0 | 0 | 21 | Good |
| Chiang^73^ | 2010 | 1 | 1 | 1 | 1 | 2 | 1 | 1 | 0 | 0 | 1 | 0 | 0 | 1 | 0 | 0 | 1 | 1 | 1 | 1 | 1 | 1 | 1 | 1 | 0 | 1 | 0 | 0 | 19 | Fair |
| Cobb^63^ | 2014 | 1 | 1 | 1 | 1 | 2 | 0 | 0 | 0 | 0 | 1 | 0 | 0 | 1 | 0 | 1 | 1 | 1 | 1 | 1 | 1 | 1 | 1 | 1 | 0 | 1 | 1 | 1 | 20 | Good |
| Cohen-Mansfield^35^ | 2018 | 1 | 1 | 1 | 1 | 2 | 1 | 1 | 0 | 0 | 0 | 0 | 0 | 1 | 0 | 0 | 1 | 1 | 1 | 1 | 1 | 1 | 1 | 1 | 0 | 1 | 1 | 0 | 19 | Fair |
| Conoley^64^ | 1985 | 1 | 1 | 1 | 1 | 0 | 1 | 1 | 0 | 1 | 0 | 0 | 0 | 1 | 0 | 0 | 1 | 1 | 1 | 1 | 1 | 1 | 1 | 1 | 0 | 0 | 1 | 0 | 17 | Fair |
| Creswell^71^ | 2012 | 1 | 1 | 1 | 1 | 2 | 1 | 1 | 0 | 0 | 1 | 1 | 1 | 1 | 0 | 1 | 1 | 1 | 1 | 1 | 1 | 1 | 1 | 1 | 0 | 1 | 1 | 0 | 23 | Good |
| Czaja^79^ | 2018 | 1 | 1 | 1 | 1 | 2 | 0 | 1 | 0 | 1 | 0 | 1 | 0 | 1 | 1 | 1 | 1 | 1 | 1 | 1 | 1 | 1 | 1 | 1 | 0 | 1 | 1 | 1 | 23 | Good |
| Dickens^42^ | 2011 | 1 | 1 | 1 | 1 | 2 | 1 | 1 | 0 | 0 | 1 | 0 | 0 | 1 | 0 | 0 | 1 | 1 | 1 | 1 | 1 | 0 | 1 | 0 | 0 | 1 | 1 | 1 | 19 | Fair |
| Dodge^65^ | 2015 | 1 | 1 | 1 | 1 | 2 | 0 | 1 | 0 | 1 | 1 | 1 | 0 | 1 | 0 | 0 | 1 | 1 | 1 | 1 | 1 | 1 | 1 | 1 | 0 | 1 | 1 | 1 | 22 | Good |
| Dowd^49^ | 2014 | 1 | 1 | 1 | 1 | 0 | 0 | 1 | 0 | 0 | 1 | 0 | 0 | 1 | 1 | 0 | 1 | 1 | 1 | 1 | 1 | 1 | 1 | 1 | 0 | 0 | 1 | 1 | 18 | Fair |
| Elsherbiny^69^ | 2018 | 1 | 1 | 1 | 1 | 2 | 0 | 1 | 0 | 0 | 0 | 1 | 1 | 1 | 0 | 0 | 1 | 1 | 1 | 1 | 1 | 1 | 1 | 1 | 0 | 0 | 0 | 0 | 18 | Fair |
| Hall^40^ | 1992 | 1 | 1 | 1 | 1 | 2 | 1 | 1 | 0 | 0 | 1 | 1 | 0 | 1 | 0 | 1 | 1 | 1 | 1 | 1 | 1 | 1 | 1 | 1 | 0 | 0 | 0 | 1 | 21 | Good |
| Heller^27^ | 1991 | 1 | 1 | 1 | 1 | 0 | 1 | 1 | 0 | 0 | 0 | 1 | 0 | 1 | 0 | 0 | 1 | 1 | 1 | 1 | 1 | 1 | 1 | 1 | 0 | 1 | 0 | 0 | 17 | Fair |
| Iliffe^51^ | 2014 | 1 | 1 | 1 | 1 | 0 | 1 | 1 | 1 | 1 | 1 | 1 | 0 | 1 | 0 | 0 | 1 | 1 | 1 | 1 | 1 | 1 | 1 | 1 | 1 | 1 | 0 | 1 | 22 | Good |
| Jessen^25^ | 1996 | 1 | 1 | 1 | 1 | 0 | 1 | 1 | 0 | 1 | 1 | 0 | 0 | 1 | 0 | 0 | 1 | 1 | 1 | 1 | 1 | 1 | 1 | 1 | 0 | 1 | 1 | 0 | 19 | Fair |
| Jung^60^ | 2009 | 1 | 1 | 1 | 1 | 0 | 1 | 1 | 0 | 0 | 0 | 0 | 0 | 1 | 0 | 0 | 1 | 1 | 1 | 1 | 1 | 1 | 1 | 1 | 0 | 0 | 0 | 0 | 15 | Fair |
| Kahlbaugh^59^ | 2011 | 1 | 1 | 1 | 1 | 1 | 0 | 1 | 0 | 1 | 0 | 0 | 0 | 1 | 0 | 0 | 1 | 1 | 1 | 1 | 1 | 1 | 1 | 1 | 0 | 1 | 1 | 0 | 18 | Fair |
| Kamegaya^50^ | 2014 | 1 | 1 | 1 | 1 | 2 | 1 | 1 | 0 | 0 | 1 | 1 | 0 | 1 | 0 | 0 | 1 | 1 | 1 | 0 | 1 | 1 | 1 | 1 | 0 | 1 | 0 | 0 | 19 | Fair |
| Kremers^36^ | 2006 | 1 | 1 | 1 | 1 | 2 | 1 | 1 | 0 | 0 | 0 | 0 | 0 | 1 | 0 | 0 | 1 | 1 | 1 | 1 | 1 | 1 | 1 | 1 | 0 | 1 | 1 | 0 | 19 | Fair |
| Kuru Alici^68^ | 2018 | 1 | 1 | 1 | 1 | 2 | 1 | 1 | 0 | 0 | 1 | 0 | 0 | 1 | 0 | 0 | 1 | 1 | 1 | 1 | 1 | 0 | 1 | 0 | 0 | 1 | 0 | 1 | 18 | Fair |
| Lokk^80^ | 1990 | 1 | 1 | 1 | 0 | 1 | 1 | 1 | 0 | 0 | 1 | 0 | 0 | 0 | 0 | 0 | 1 | 1 | 1 | 1 | 0 | 1 | 1 | 1 | 0 | 0 | 0 | 0 | 14 | Poor |
| Maki^52^ | 2012 | 1 | 1 | 1 | 1 | 2 | 1 | 1 | 0 | 0 | 1 | 1 | 0 | 1 | 0 | 0 | 1 | 1 | 1 | 0 | 1 | 1 | 1 | 1 | 0 | 1 | 0 | 0 | 19 | Fair |
| Martina^32^ | 2006 | 1 | 1 | 1 | 1 | 2 | 1 | 1 | 0 | 0 | 1 | 0 | 0 | 1 | 0 | 0 | 1 | 1 | 1 | 1 | 1 | 0 | 0 | 0 | 0 | 0 | 0 | 0 | 15 | Fair |
| McAuley^53^ | 2000 | 1 | 1 | 1 | 1 | 2 | 0 | 1 | 0 | 0 | 0 | 0 | 0 | 1 | 0 | 0 | 1 | 1 | 0 | 1 | 1 | 1 | 1 | 1 | 0 | 1 | 1 | 0 | 17 | Fair |
| McWhirter^66^ | 1996 | 1 | 1 | 1 | 1 | 0 | 1 | 1 | 0 | 0 | 1 | 0 | 0 | 1 | 0 | 0 | 1 | 1 | 1 | 1 | 1 | 1 | 1 | 1 | 0 | 0 | 0 | 1 | 17 | Fair |
| Mountain^28,29^ | 2014 | 1 | 1 | 1 | 1 | 2 | 1 | 1 | 0 | 0 | 0 | 1 | 0 | 1 | 0 | 0 | 1 | 1 | 1 | 1 | 1 | 1 | 1 | 1 | 1 | 1 | 1 | 1 | 22 | Good |
| Mountain^37^ | 2017 | 1 | 1 | 1 | 1 | 2 | 1 | 1 | 0 | 0 | 1 | 0 | 0 | 1 | 0 | 1 | 1 | 1 | 1 | 1 | 1 | 1 | 1 | 1 | 0 | 1 | 0 | 1 | 21 | Good |
| Mutrie^54^ | 2012 | 1 | 1 | 1 | 1 | 2 | 0 | 0 | 0 | 0 | 0 | 1 | 1 | 1 | 0 | 1 | 1 | 1 | 1 | 1 | 1 | 1 | 1 | 1 | 1 | 1 | 0 | 0 | 20 | Good |
| Perkins^56^ | 2012 | 1 | 1 | 1 | 1 | 0 | 1 | 1 | 0 | 0 | 1 | 0 | 0 | 1 | 0 | 0 | 1 | 1 | 1 | 1 | 1 | 0 | 1 | 0 | 0 | 0 | 0 | 0 | 14 | Poor |
| Robinson^26^ | 2013 | 1 | 1 | 1 | 1 | 0 | 1 | 1 | 0 | 0 | 1 | 0 | 0 | 1 | 0 | 0 | 1 | 1 | 1 | 1 | 1 | 1 | 1 | 1 | 0 | 1 | 0 | 0 | 17 | Fair |
| Ruffing-Rahal^38^ | 1994 | 1 | 1 | 1 | 1 | 2 | 1 | 1 | 0 | 1 | 1 | 0 | 0 | 1 | 0 | 0 | 1 | 1 | 1 | 1 | 1 | 0 | 1 | 0 | 0 | 1 | 1 | 0 | 19 | Fair |
| Saito^81^ | 2012 | 1 | 1 | 1 | 1 | 2 | 1 | 1 | 0 | 0 | 1 | 1 | 0 | 1 | 0 | 0 | 1 | 1 | 1 | 1 | 1 | 1 | 1 | 1 | 1 | 1 | 1 | 0 | 22 | Good |
| Schulz^30^ | 1976 | 1 | 1 | 1 | 1 | 0 | 1 | 0 | 0 | 0 | 0 | 0 | 0 | 1 | 0 | 1 | 1 | 1 | 1 | 1 | 0 | 1 | 1 | 1 | 0 | 0 | 0 | 0 | 14 | Poor |
| Seepersad^39^ | 2005 | 1 | 1 | 1 | 1 | 2 | 1 | 0 | 0 | 0 | 0 | 0 | 0 | 1 | 0 | 0 | 1 | 1 | 1 | 1 | 1 | 0 | 1 | 0 | 0 | 0 | 1 | 0 | 15 | Fair |
| Shapira^43^ | 2007 | 1 | 1 | 1 | 1 | 2 | 1 | 1 | 0 | 0 | 0 | 0 | 0 | 1 | 0 | 0 | 1 | 1 | 1 | 1 | 1 | 0 | 1 | 0 | 0 | 1 | 1 | 0 | 17 | Fair |
| Slegers^44^ | 2008 | 1 | 1 | 1 | 1 | 0 | 1 | 1 | 0 | 0 | 1 | 0 | 0 | 1 | 0 | 0 | 1 | 1 | 1 | 1 | 1 | 0 | 1 | 0 | 0 | 1 | 0 | 1 | 16 | Fair |
| Tarazona-Santabalbina^55^ | 2016 | 1 | 1 | 1 | 1 | 2 | 1 | 1 | 0 | 0 | 1 | 1 | 0 | 1 | 0 | 1 | 1 | 1 | 1 | 1 | 1 | 1 | 1 | 1 | 0 | 1 | 1 | 1 | 23 | Good |
| Tsai^82^ | 2010 | 1 | 1 | 1 | 1 | 2 | 1 | 1 | 0 | 0 | 1 | 1 | 0 | 1 | 0 | 0 | 1 | 1 | 1 | 1 | 1 | 0 | 1 | 0 | 0 | 1 | 0 | 0 | 18 | Fair |
| Tsai^83^ | 2011 | 1 | 1 | 1 | 1 | 2 | 1 | 1 | 0 | 0 | 1 | 1 | 0 | 1 | 0 | 0 | 1 | 1 | 1 | 1 | 1 | 0 | 1 | 1 | 0 | 1 | 1 | 0 | 20 | Good |
| Tse^57^ | 2010 | 1 | 1 | 1 | 1 | 2 | 1 | 1 | 0 | 0 | 1 | 0 | 0 | 1 | 0 | 0 | 1 | 1 | 1 | 1 | 1 | 0 | 1 | 0 | 0 | 1 | 0 | 0 | 17 | Fair |
| Tilburg^34^ | 2000 | 1 | 1 | 1 | 1 | 1 | 1 | 1 | 0 | 1 | 1 | 1 | 0 | 1 | 0 | 0 | 1 | 0 | 1 | 1 | 1 | 0 | 0 | 0 | 0 | 0 | 1 | 0 | 16 | Fair |
| van Rossum^41^ | 1993 | 1 | 1 | 1 | 1 | 2 | 0 | 0 | 0 | 0 | 0 | 0 | 0 | 1 | 0 | 1 | 1 | 1 | 1 | 1 | 1 | 1 | 1 | 1 | 0 | 1 | 0 | 0 | 17 | Fair |
| Westerhof^74^ | 2018 | 1 | 1 | 1 | 1 | 0 | 1 | 1 | 0 | 0 | 1 | 0 | 0 | 1 | 0 | 0 | 1 | 1 | 1 | 1 | 1 | 1 | 1 | 1 | 0 | 1 | 1 | 1 | 19 | Fair |
| White^45^ | 1999 | 1 | 1 | 0 | 1 | 2 | 0 | 1 | 0 | 0 | 1 | 0 | 0 | 1 | 0 | 0 | 1 | 1 | 1 | 1 | 1 | 1 | 1 | 0 | 0 | 1 | 0 | 0 | 16 | Fair |
| White^46^ | 2002 | 1 | 1 | 0 | 1 | 2 | 0 | 1 | 0 | 0 | 1 | 0 | 0 | 1 | 0 | 0 | 1 | 1 | 1 | 1 | 1 | 1 | 1 | 1 | 0 | 1 | 0 | 0 | 17 | Fair |
| Wikstrom^76^ | 2002 | 1 | 1 | 1 | 1 | 0 | 1 | 0 | 0 | 0 | 1 | 0 | 0 | 0 | 0 | 0 | 1 | 1 | 1 | 1 | 0 | 1 | 1 | 1 | 0 | 0 | 0 | 0 | 13 | Poor |
| Winningham^67^ | 2007 | 1 | 1 | 0 | 1 | 0 | 1 | 1 | 0 | 0 | 1 | 0 | 0 | 1 | 0 | 0 | 1 | 1 | 1 | 1 | 1 | 0 | 1 | 0 | 0 | 0 | 0 | 0 | 13 | Poor |
| Winstead^62^ | 2014 | 1 | 1 | 1 | 1 | 2 | 1 | 1 | 0 | 1 | 0 | 0 | 0 | 1 | 0 | 0 | 1 | 1 | 1 | 1 | 1 | 0 | 1 | 1 | 0 | 1 | 1 | 0 | 19 | Fair |
| Woodward^47^ | 2011 | 1 | 1 | 1 | 1 | 2 | 0 | 0 | 0 | 0 | 0 | 0 | 0 | 1 | 0 | 0 | 1 | 1 | 1 | 1 | 1 | 1 | 1 | 1 | 0 | 1 | 0 | 0 | 16 | Fair |
| Xu^61^ | 2016 | 1 | 1 | 1 | 1 | 2 | 0 | 1 | 0 | 0 | 1 | 0 | 0 | 1 | 0 | 0 | 1 | 1 | 1 | 1 | 1 | 0 | 1 | 0 | 0 | 0 | 0 | 0 | 15 | Fair |
| Zhang^72^ | 2018 | 1 | 1 | 1 | 1 | 1 | 1 | 1 | 0 | 0 | 1 | 1 | 0 | 1 | 0 | 0 | 1 | 1 | 1 | 1 | 1 | 1 | 1 | 1 | 0 | 1 | 1 | 0 | 20 | Good |
| **Non-feasible Interventions** | | | | | | | | | | | | | | | | | | | | | | | | | | | | | | |
| Alaviani^8^ | 2015 | 1 | 1 | 1 | 1 | 2 | 1 | 1 | 0 | 0 | 1 | 0 | 0 | 1 | 0 | 0 | 1 | 1 | 1 | 1 | 1 | 1 | 1 | 1 | 0 | 1 | 0 | 0 | 19 | Fair |
| Banks^1^ | 2002 | 1 | 1 | 1 | 1 | 0 | 0 | 1 | 0 | 0 | 1 | 0 | 0 | 1 | 0 | 0 | 1 | 1 | 1 | 1 | 1 | 1 | 1 | 1 | 0 | 0 | 0 | 1 | 16 | Fair |
| Banks^2^ | 2005 | 1 | 1 | 1 | 1 | 2 | 0 | 1 | 0 | 0 | 0 | 0 | 0 | 1 | 0 | 0 | 1 | 1 | 1 | 1 | 1 | 1 | 1 | 1 | 0 | 0 | 0 | 1 | 17 | Fair |
| Boen^23^ | 2012 | 1 | 1 | 1 | 1 | 2 | 1 | 1 | 0 | 0 | 0 | 1 | 0 | 1 | 0 | 0 | 1 | 1 | 1 | 1 | 1 | 1 | 1 | 1 | 0 | 0 | 0 | 0 | 18 | Fair |
| Clarke^13^ | 1992 | 1 | 1 | 1 | 1 | 1 | 0 | 1 | 0 | 1 | 1 | 1 | 0 | 1 | 0 | 1 | 1 | 1 | 1 | 1 | 1 | 1 | 1 | 1 | 0 | 1 | 1 | 1 | 22 | Good |
| Cohen^19^ | 2006 | 1 | 1 | 1 | 1 | 2 | 1 | 1 | 0 | 0 | 0 | 0 | 0 | 1 | 0 | 0 | 1 | 1 | 1 | 0 | 1 | 1 | 1 | 0 | 0 | 1 | 0 | 0 | 16 | Fair |
| de Craen^11^ | 2006 | 1 | 1 | 1 | 1 | 2 | 0 | 1 | 0 | 1 | 1 | 1 | 0 | 1 | 1 | 0 | 1 | 1 | 1 | 1 | 1 | 1 | 1 | 1 | 1 | 1 | 1 | 1 | 24 | Good |
| Honigh – de Vlaming^9^ | 2013 | 1 | 1 | 1 | 1 | 2 | 1 | 1 | 0 | 0 | 1 | 1 | 0 | 1 | 0 | 0 | 1 | 1 | 1 | 1 | 1 | 1 | 1 | 1 | 0 | 1 | 1 | 1 | 22 | Good |
| Lupton^10^ | 2005 | 1 | 1 | 1 | 1 | 1 | 1 | 0 | 0 | 0 | 1 | 1 | 0 | 1 | 0 | 0 | 1 | 1 | 1 | 0 | 0 | 0 | 1 | 0 | 0 | 1 | 0 | 0 | 14 | Poor |
| MacIntyre^5^ | 1999 | 1 | 1 | 1 | 1 | 2 | 0 | 1 | 0 | 0 | 1 | 1 | 0 | 1 | 0 | 0 | 1 | 1 | 1 | 1 | 1 | 1 | 1 | 1 | 0 | 1 | 0 | 0 | 19 | Fair |
| McEwan^14^ | 1990 | 1 | 1 | 1 | 1 | 1 | 1 | 0 | 0 | 0 | 0 | 1 | 0 | 1 | 0 | 0 | 1 | 1 | 1 | 1 | 1 | 1 | 1 | 1 | 0 | 0 | 1 | 1 | 18 | Fair |
| Melis^12^ | 2008 | 1 | 1 | 1 | 1 | 2 | 0 | 1 | 0 | 0 | 0 | 0 | 0 | 1 | 0 | 1 | 1 | 1 | 1 | 1 | 1 | 1 | 1 | 1 | 1 | 1 | 1 | 0 | 20 | Good |
| Morrow-Howell^15^ | 1998 | 1 | 1 | 1 | 1 | 1 | 1 | 0 | 0 | 0 | 1 | 1 | 0 | 1 | 0 | 1 | 1 | 1 | 1 | 1 | 1 | 1 | 1 | 1 | 0 | 0 | 1 | 0 | 19 | Fair |
| Mulligan^6^ | 1978 | 1 | 1 | 1 | 1 | 1 | 1 | 1 | 0 | 1 | 0 | 0 | 0 | 1 | 1 | 0 | 1 | 1 | 0 | 1 | 1 | 0 | 1 | 0 | 0 | 0 | 0 | 0 | 15 | Fair |
| Ollonqvist^16^ | 2008 | 1 | 1 | 1 | 1 | 2 | 1 | 1 | 0 | 0 | 0 | 1 | 0 | 1 | 0 | 1 | 1 | 1 | 1 | 1 | 0 | 1 | 1 | 1 | 1 | 1 | 1 | 1 | 22 | Good |
| Parsons^17^ | 2013 | 1 | 1 | 1 | 1 | 2 | 1 | 1 | 0 | 1 | 1 | 1 | 0 | 1 | 0 | 1 | 1 | 1 | 1 | 1 | 1 | 1 | 1 | 1 | 1 | 1 | 1 | 1 | 25 | Good |
| Pynnonen^21^ | 2018 | 1 | 1 | 1 | 1 | 2 | 1 | 1 | 0 | 0 | 1 | 1 | 0 | 1 | 0 | 1 | 1 | 1 | 1 | 1 | 0 | 1 | 1 | 1 | 0 | 1 | 1 | 0 | 21 | Good |
| Riddick^3^ | 1985 | 1 | 1 | 1 | 1 | 1 | 1 | 1 | 0 | 0 | 1 | 0 | 0 | 1 | 0 | 0 | 1 | 1 | 1 | 1 | 1 | 1 | 1 | 0 | 0 | 0 | 0 | 0 | 16 | Fair |
| Rook^7^ | 2003 | 1 | 1 | 1 | 1 | 2 | 1 | 1 | 0 | 0 | 0 | 1 | 0 | 1 | 0 | 1 | 1 | 1 | 1 | 1 | 1 | 0 | 0 | 0 | 0 | 1 | 1 | 1 | 19 | Fair |
| Routasalo^22^ | 2009 | 1 | 1 | 1 | 1 | 2 | 0 | 1 | 0 | 0 | 0 | 1 | 0 | 1 | 0 | 0 | 1 | 1 | 1 | 1 | 1 | 1 | 1 | 1 | 1 | 1 | 0 | 0 | 19 | Fair |
| Sollami^4^ | 2017 | 1 | 1 | 1 | 1 | 1 | 1 | 1 | 0 | 1 | 1 | 0 | 0 | 1 | 0 | 0 | 1 | 1 | 1 | 1 | 1 | 1 | 1 | 1 | 0 | 0 | 1 | 1 | 20 | Good |
| Tse^18^ | 2014 | 1 | 1 | 1 | 1 | 2 | 1 | 1 | 0 | 0 | 1 | 0 | 0 | 1 | 0 | 0 | 1 | 1 | 1 | 1 | 1 | 1 | 1 | 1 | 0 | 1 | 0 | 0 | 19 | Fair |
| Yap^20^ | 2017 | 1 | 1 | 1 | 1 | 2 | 1 | 1 | 1 | 0 | 1 | 0 | 0 | 1 | 0 | 0 | 1 | 1 | 1 | 1 | 1 | 1 | 1 | 1 | 1 | 1 | 1 | 1 | 23 | Good |

**S4 Table:** Summary characteristics of the main systematic reviews from which included primary studies were retrieved, once duplicates and non-eligible reviews were excluded.

| **Systematic Review** | **Search Range** | **Databases Searched** | **Key Search Terms** | **Inclusion/Exclusion Criteria** |
| --- | --- | --- | --- | --- |
| Abbott  (2019)^84^ | Inception - 2018 | MEDLINE, EMBASE, PsycINFO, SPP, CINAHL, AgeLine, CDSR, CENTRAL, DARE, ASSIA, Web of Science, Scopus, and ProQuest Dissertations and Thesis Global.  (n = 13) | Animal*, pet*, robo*, bonding, human-pet, animal assisted therapy, nursing, geriatric*, adult, old*, senior*, elder*, aged*, dement*, Alzheimer*, institut*, resident*, unit*, facilit*, home*, care home*, long-term care, resident* care, home nursing and home(s) for the aged. | Inclusion:  1. Robopets defined as small animal-like robots with behavioural characteristics of a pet.  2. Articles reported the views and experiences of robopet interaction of care home residents, their families and care staff, or;  3. Articles reported the effects of robopets on health and well-being, social interaction, physical function, behaviour, or medication use.  4. Qualitative or quantitative studies.  Exclusion:  1. Excluded if did not meet inclusion criteria. |
| Baker  (2018)^85^ | 2000 - 2016 | Scopus, Compendex, Inspec, Association for Computing Machinery and Web of Science.  (n = 5) | Information and communication technology, ICT, Information systems, Human-computer interaction, HCI, seniors, elderly, older people and social. | Inclusion:   1. 1. Articles had been peer reviewed.  2. Technology referred to ICTs and could include devices, applications and websites.  3. The term older people was as used in the literature.  4. Articles included the use of any technology targeted at older people aimed at supporting social participation/reducing isolation.  5. Empirical research   Exclusion:  1. Excluded terms during search: Organisation, Management, Medical Information Systems, Adolescents, Telemedicine, Young Adults, Robot*, Geographic Information System*, GIS, Information Management.  2. Studies were theoretical or descriptive studies. |
| Bessaha  (2020)^86^ | Not specified | PsycINFO, CINAHL, PubMed and Social Work Abstracts.  (n = 4) | The search used standardised terms and keywords related to loneliness, social interventions and non-elderly adults – no further detail is given in the article or supplementary data. | Inclusion:   1. 1. Articles described or evaluated an intervention targeting loneliness in non-elderly adults.   Exclusion:  1. Studies in which the endpoint of an intervention was something other than loneliness.  2. Studies in which the average age of participants was greater than 65 or under 18. |
| Cattan  (2005)^87^ | 1970 - 2002 | MEDLINE, SCI, SSCI, EMBASE, PsycINFO, ASSIA, CINAHL, SweMed, FirstSearch, Academic Search Elite, SIGLE, the Cochrane Library, and LILACS.  (n = 13) | Older*, elder*, senior*, geriatric, aged, social isolation, isolation, loneliness, social, social support, loss, promot*, prevent*, support, self-help, review, overview, evaluation, intervention. Further peripheral search terms specified in primary article. | Inclusion:   1. 1. The target population of the study was older people.  2. Studies in which the intervention intended to alleviate social isolation and/or loneliness.  3. Studies evaluated health-promoting interventions that helped older people increase their control over their health.  4. Studies reported outcome measures with or without process measures.   Exclusion:   1. 1. Excluded if did not meet inclusion criteria. |
| Cohen-Mansfield (2015)^88^ | 1996 - 2011 | PsycINFO, MEDLINE, ScienceDirect, AgeLine, PsycBOOKS, and Google Scholar. (n = 6) | Loneliness, social isolation, isolation, older adults, older, elderly, senior, geriatric, aged, social support, and loss, community program, community development, empower, education, technology, neighbourhood, depression, suicide, information, psychosocial, and social activity | Inclusion:   1. 1. Study population was older adults, 55 years old or older.  2. Studies which implemented or examined an intervention's effect on loneliness.  3. Study outcome measures included changes in level of loneliness or loneliness-related measures such as social interaction or social initiative.  4. Studies in which pre-test and post-test comparisons were made.   Exclusion:  1. Any studies which didn't report inferential statistics unless they used an innovative intervention tactic.  2. Studies which didn't make pre-test and post-test comparisons. |
| Coll-Planas  (2017)^89^ | 1980 - 2015 | MEDLINE, EMBASE, CINAHL, PsycINFO, the Cochrane Library and the Web of Science. (n = 6) | Aging, nursing home*, long-term care, caregiver(s), homebound persons, home care services, old(s), senior(s), ageing, aged, community dwelling, care home*, carer(s), resident, geriatrics, elderly, elder, elders, social capital, social network, social support, social/ political/ civic participation, social activit*, leisure activit*, institutional trust, psychosocial rehabilitation, social cohesion, interpersonal relation*, social relation*, social ties, social contact, psychosocial support, peer support, peer advisor, psychosocial intervention/ program, befriend, friends, friendship, social interaction, sense of belonging, social engagement, social exclusion, social isolation, community network, randomised controlled trial, controlled clinical trial, trial. | Inclusion:   1. 1. Studies that were randomised controlled trials.  2. Studies with a target population aged over 60.  3. Studies assessed an intervention that promoted social capital or one of its components.  4. Studies reported effects on health outcomes or use of health-related resources.   Exclusion:  1. Studies which employed professional support as this was not considered social support and hence not social capital.  2. Studies in which the comparison group contained social capital elements. |
| Dickens  (2011)^90^ | Inception - 2009 | MEDLINE, EMBASE, ASSIA, IBSS, PsycINFO, PubMed, DARE, Social Care Online, the Cochrane Library and CINAHL.  (n = 10) | Befriend*, home visit*, visit* program*, mentor*, psychosocial, network*, prevent*, promot*, support, self-help, social* activ*, health promotion, health status, community network, social* participat*, social* integrat*, friendship, quality of life, wellbeing, self-esteem, creative activ*, exercise, physical active*, peer, socio-medical | Inclusion:   1. 1. The target population of the study was older adults.  2. The intervention targeted those who were socially isolated and/or lonely and aimed to improve this.  3. Studies reported outcome measures for participants with sufficient data for treatment effects to be analysed.  4. Studies were randomised controlled trials or quasi-experimental.  5. Studies had a control group.   Exclusion:   1. 1. Excluded if did not meet inclusion criteria. |
| Forsman  (2018)^91^ | 2003 - 2014 | MEDLINE, PsycINFO, Ageline, ASSIA, ERIC, Social Care Online and DARE.  (n = 7) | Aged, retirement, elder*, frail*, geriatric*, gerontology, seniors, senior citizen*, retire*, pensioner*, later-life, late life, old age, old* people, old* person, old* adult, psychological resilience, psychological adaptation, social support, social distance, community networks, independent living,, quality of life, social identification, happiness, mental health, personal satisfaction, emotional health, emotional capital, mental capital, wellness, wellbeing, activities of daily living, healthy aging, active aging, meaningfulness, loneliness, mastery, locus of control, capability*, empower*, social capital, social relation, family relation, participation, social contact, psychosocial, social inclusion, social exclusion, independence, dignity, choice, isolation, intervention*, initiative*, program*, promot*, social media, communication, health promotion, family, friends, residential facilities, nursing homes, residential care, long term care, palliative care | Inclusion:   1. 1. Studies focused on individuals who did not have any significant health or social care needs.  2. Studies in which the participants were living independently in the community.  3. Studies with interventions that were technology-based and promoted mental well-being or independence.  4. Studies with interventions that promoted the mental well-being of spousal carers, relatives and friends of older people.  5. Study populations must be aged over 65 or over 55 and retired.   Exclusion:  1. Studies in which interventions were delivered by health and social care professionals. |
| Gee  (2019)^92^ | 1965 - 2018 | Academic Search Complete, Alt Health Watch, Google Scholar, CINAHL, Education Source, Anthrozoӧs, Environment Complete, ERIC, Health Source: Nursing/Academic Edition, Waltham.com, LGBT Life, HABRI, MEDLINE, PsychARTICLES, Psychology and Behavioral Sciences Collection, PsycINFO, Social Sciences and SocINDEX.  (n = 18) | Older adults, elderly, seniors, geriatrics, aging, age related, aged, companion animals, pets, dogs, canine, feline, cats, fish, birds, pet ownership, animal interaction, animal assisted therapy, animal assisted activities animal assisted interventions, animal assisted education. | Inclusion:   1. 1. Articles that had been peer reviewed.  2. Articles written in English.  3. Articles which presented original research on human-animal interaction in older adults (defined as 50 years old and above.)   Exclusion:   1. 1. Excluded if did not meet inclusion criteria. |
| Hagan  (2014)^93^ | 2000 - 2012 | PsycINFO, MEDLINE, CINAHL, ASSIA, Scopus, Social Services Abstracts and Sociological Abstracts.  (n = 7) | Old*, eld*, geri*, loneliness, social isolation, social network, social support. | Inclusion:   1. 1. Articles that had been peer-reviewed.  2. Articles written in English.  3. Articles with human participants.   Exclusion:   1. 1. Excluded if did not meet inclusion criteria. |
| Kall  (2020)^94^ | Not specified | Scopus and PsycINFO.  (n = 2) | Lonel*, treat*, interv* on Scopus and lonel*, interven*, treat* on PsycINFO | Inclusion:   1. 1. Studies with an intervention seeking to change the participants’ behavioural and cognitive responses and/or coping strategies with the intention of reducing loneliness.  2. Studies in which the intervention group compare favourably to a control group in a randomised study.  3. Studies using a quantitative scale to measure loneliness as a primary outcome measure.   Exclusion:  1. Interventions that focused on altering environmental aspects for participants were excluded (e.g. animal support therapy or social support through a volunteer program). |
| Li  (2018)^95^ | Inception - 2017 | PsycINFO, PubMed, CINAHL and ScienceDirect.  (n = 4) | Exergame, Wii, Kinect, active video game, socia*, social support, social interaction, social bonding, communicat*, aging, aged, elderly, older, senior. | Inclusion:   1. 1. Studies in which the primary intervention involved exergames.  2. Studies reported outcome measures of social outcomes such as social connection, social bonding or loneliness.  3. Studies with a target population aged 55 or above.  4. Articles written in English.  5. Articles reporting original research.  6. Quantitative and qualitative studies.   Exclusion:   1. 1. Excluded if did not meet inclusion criteria. |
| Masi  (2011)^96^ | 1970 - 2009 | PubMed and PsycINFO.  (n = 2) | Loneliness, intervention, treatment, prospective, medication, and pharmacology. | Inclusion:   1. 1. The intervention directly targeted loneliness.  2. The effect of the intervention was measured and reported quantitatively to enable calculation of effect size.  3. Studies reported original research not reported in another article.  4. The intervention involved a treatment group and not just individual cases.   Exclusion:   1. 1. Excluded if did not meet inclusion criteria. |
| Mikkelsen  (2019)^97^ | Inception - 2017 | PubMed, Scopus and PsycINFO.  (n = 3) | Social relations, social support, interpersonal relation, interpersonal relations, psychology, social, social identity, social identities, sense of belonging, social isolation, loneliness, unwanted, alone, social network(s), social interaction(s), intervention, community intervention, community-based intervention, group-based intervention, implementation, social planning, psychosocial intervention, guideline(s), intervention study, intervention studies, evaluation study, evaluation studies, multicomponent intervention(s), social activity, social activities, aging, ageing, aged, older adults, elder, elderly, older people, old age, nursing home, home(s) for the aged, old age home(s), nursing home(s), assisted living facility, assisted living facilities, retirement home(s), old people’s home(s), daily care, 24-hour care, care home(s). | Inclusion:   1. 1. Studies in which the base theory of the intervention or the outcome measures of the intervention focused on social relations or related terms such as loneliness, social support, social isolation or social network.  2. Studies in which the interventions were implemented in nursing homes or similar settings.  3. The intervention was a narrative activity.  4. Studies in which the participants met physically or non-physically.  5. Studies targeted the residents of care home and not the employees or relatives.   Exclusion:   1. 1. Excluded if did not meet inclusion criteria. |
| Nnabuko  (2018)^98^ | 2000 - 2017 | PubMed, clinicaltrials.gov and the Cochrane Library.  (n = 3) | Social support, ICT, social media, social network, telemedicine, telecare, telehealth, tele* and online games. | Inclusion:   1. 1. Articles written in English.  2. Studies reported quantitative data.  3. Studies evaluated the effects of an ICT intervention on a health and care related condition.  4. Studies in which the intervention was administered to more than a single participant.  5. Studies in which social support was measured as an independent variable.   Exclusion:   1. 1. Excluded if did not meet inclusion criteria. |
| Pels  (2016)^99^ | Inception - 2015 | PsycINFO, PsycARTICLES, PSYNDEX and SPORTDiscus.  (n = 4) | Physical* activ*, sport*, exercis*, lonel*. | Inclusion:   1. 1. Articles had been peer-reviewed.  2. Articles written in English.  3. Articles reported original research.  4. Studies examined the relationship between loneliness and physical activity.   Exclusion:  1. Articles focused explicitly on social isolation and not on loneliness. |
| Poscia  (2018)^100^ | 2011 - 2016 | PubMed, Web of science, Scopus, the Cochrane Library and CINAHL.  (n = 5) | Social isolation, solitude, aloneness, loneliness, emotional isolation, older, elder*, senior*, geriatric, aged, ageing, ageing, old age old people, social participation, social support, social involvement, promotion, program, programme, plan, intervention, health promotion, prevention, campaign, health programme” health program, health prevention, social care, social intervention, screening, health education, health literacy, health communication, health advocacy, community advocacy, social campaign, social campaigns, health coaching, environmental change strategies, healthy environment, community mobilization, behaviour modification, screening, primary prevention, health screening, support groups, social network, social gathering, health changes, legislation, regulation, effectiveness, efficacy, efficiency, impact, evidence, outcomes. | Inclusion:   1. 1. Articles written in English or Italian.  2. Studies evaluated an intervention that targeted loneliness or social isolation.  3. Studies in which the target population was older than 65 or specifically targeted older people.  4. Studies which reported outcome measures for social isolation or loneliness, using quantitative and qualitative study designs.   Exclusion:   1. 1. Excluded if did not meet inclusion criteria. |
| Quan  (2019)^101^ | 2009 - 2019 | PubMed, PsycINFO and Web of Science.  (n = 3) | Senior*, retire*, older, old, late life, geriatric*, septuagenarian*, octogenarian*, nonagenarian*, aging, ageing, elder*, aged, loneliness, social isolation, lonely, social alienation, socially isolated, socially alienated, assisted living facilities, group home*, homes for the aged, institutionalisation, nursing home*, assisted living, care home*, centre(s), extended care, old age home*, residential care, rest home*, retirement communit*, retirement home* | Inclusion:   1. 1. Articles written in English.  2. The study evaluated an intervention with a quantitative comparison of pre-trial and post-trial measures.  3. Loneliness was one of the primary outcome measures.  4. More than half of participants were aged 65 or older.  5. Participants live in a facility such as a nursing home, assisted living or a hospice.   Exclusion:  1. Facilities defined as a place where participants received some form of healthcare or functional support for a duration of one month or longer. Hence, acute care hospitals or facilities which did not provide healthcare or functional support were excluded. |
| Shvedko  (2018)^102^ | Inception - 2017 | MEDLINE, EMBASE, PsycINFO, the Cochrane Library and CINAHL.  (n = 5) | Physical activity, exercise, loneliness, social isolation, social support, social network, quality of life, aged, elderly | Inclusion:   1. 1. Study population was community dwelling adults aged 60 or above that were mobile and healthy or with comorbidities.  2. The study interventions were physical activity-based and could be gym-based, home-based, community-based or telephone-based.  3. Studies compared a physical activity intervention group with a control group.  4. Study outcomes reported social outcomes such as loneliness, social isolation, social support, social networks or social functioning.  5. Studies were randomised controlled trials.   Exclusion:   1. 1. Excluded if did not meet inclusion criteria. |
| Siette  (2017)^103^ | Inception - 2017 | MEDLINE, EMBASE, PsycINFO, the Cochrane Library, CINAHL, Web of Knowledge, BI, Web of Science and Google Scholar.  (n = 9) | Befriend*, companion, friend, lay helper, compeer, peer, buddy, unpaid carer, informal caregiver, voluntary caregiver, naturalistic support, supported socialisation, psychosocial support, supported friendship, peer assistance, intentional friendship, consumer run services, consumers as providers, consumers-as-providers, community support, community services, paraprofessional*, nonprofessional volunteer*, nonprofessional worker*, citizen participation, civic participation, program, voluntary, helping others, supported socialization, mental health, mental illness, mental problem, mental disorder, mental health scheme, mental health charity, mental health project, mental health program*, mental health organisation, mental health service, mental health care, psychiatry, psychiatric scheme, psychiatric charity, psychiatric project, psychiatric program*, psychiatric organisation, psychiatric service, psychiatric care, psychosis, schizophrenia, severe mental illness, depression, anxiety, disorder, eating disorder, phobia, end of life care, palliative care, palliative, dementia, dementia care*, physical disabilities, HIV, AIDS, cancer, diabetes, heart failure, alcohol*, drug abuse, obsessive compulsive disorder, autism, health condition, health, physical, motivation*, motive*, reason*, opinion*, attitude*, experience*, reward*, benefit*, success*, drawback*, negative*,  positive*, ‘failure*, challenge*, difficult*, altruistic, psychological health, functioning, happiness, satisfaction, self-esteem, empowerment, well-being, outcome*. | Inclusion:   1. 1. Randomised controlled trials that compared befriending with usual care or no treatment for both physical and mental health areas.  2. Participants could be of any age. 3. Participants resided in the community and were allocated to a befriending intervention.   Exclusion:  1. Studies where informational, instructional or appraisal support formed a key component of the intervention.  2. Studies in which the befriending sessions were not delivered by volunteers or they were not offered as a free service but delivered by paid professionals.  3 Studies in which befriending was used as the control intervention.  4. Studies in which the volunteer was a member of the patients social or care network.  5. Studies in which the volunteer had experienced the same conditions as the patient. |
| Sims-Gould  (2017)^104^ | Inception - 2016 | MEDLINE, EMBASE, PsycINFO, CINAHL, SPORTDiscus, and the Cochrane Library.  (n = 6) | Ageing, aging, aged, 80 and over, aged, older, older people, older adult, senior, frail elderly, elderly, health services for the aged, home care, home care services, home support, home, community, catchment area (health), independent living, community health services, community health care, community health nursing, community nursing, home care, home visit, house calls, domiciliary care, community dwelling, community setting, homed based, restorative home, re-ablement, rehabilitation, exercise therapy, motion therapy, muscle stretching exercises, plyometric exercise, resistance training, myofunctional therapy, occupational therapy, recreation therapy, vocational, reactivation program*/ intervention, restorative care, physical activity, rehab*, activit* of daily living, re-abl*, reabl*, enablement, empower*, restor*, re-learn*, relearn*, recover*/ optim*/ maintain*/ develop*/ improv* independent ability/ outcome/ function/ living, self-care, service-use, health care cost, health services/resources, health resource utilisation, lifestyle, balance, postural balance, hand strength, muscle strength dynamometer, muscle strength, activities of daily living, gait, gait speed, randomised controlled trial, controlled clinical trial, randomised, pre-post. | Inclusion:  1. Study population was community dwelling adults aged 65 and above and may or may not be in receipt of home care.  2. Studies that delivered a Reablement, Reactivation, Rehabilitation and Restorative (4R) intervention in the home setting. 3. Studies in which group of home care recipients were not receiving 4R intervention.  4. Studies with a range of primary outcomes such as functional abilities, gait speed, social support, loneliness and ability to perform activities of daily living.  Exclusion:  1. Studies with a study population of residents in long term care.  2. Studies that examined rehabilitation or other 4R interventions delivered in hospitals, rehabilitation clinics, community centres.  3. Studies in which the intervention duration was longer than 6 months.  4. Studies that were observational, naturalistic and cross-sectional in design. |
| Veazie  (2019)^105^ | 2013 - 2018 | PubMed, PsycInfo and CINAHL.  (n = 3) | Social isolation, loneliness, social capital, social interaction, social networks, social support, communit*/ social* alienat*/ connect*/ cooperat*/ engag*/ exchang*/ exclusion/ exclude*/ include*/ inclusion/ integrat*/ interaction*/ involve*/ isolate*/ network*/ participat*/ relation*/ support*, aging, aged, attitudes towards aged, attitudes towards aging, geriatric psychiatry, geriatric psychotherapy, geriatrics, gerontology, retirement, assisted living, community-dwelling, community-residing, elder*, geriatric*, gerontol*, program evaluation, mental health program evaluation, initiative*, intervention*, innovate*, partnering, partnership*, pilot*, project*, program*, carer*, caregiver*, case manag*, employe*, family, families, student*, prison*, worker*, workplace*, cancer*, dementia, diabetes, hepatitis C, HIV, Parkinson*, psychosis, psychotic, schizophren*, app(s), computer*, internet, phone, online, videoconference* | Inclusion:   1. 1. The study population was community dwelling older people (Medicare or aged 60 and above.)  2. Studies examined an intervention that targeted social isolation to improve health outcomes.  3. Studies compared the intervention group to a control group or those which had no control group.  4. Studies assessed physical and mental health outcomes, social isolation loneliness and healthcare utilisation.  5. Studies in the community setting.  6. Studies were quantitative or mixed methods design.  7. Articles written in English.   Exclusion:  1. Study population was aged less than 60 years old.  2. Studies focused exclusively on a single health condition. 3. Studies focused on a specific subgroup of a population.  4. Studies in which the intervention was case management delivered by a trained healthcare professional, ICT-based interventions, telehealth interventions or interventions that didn’t aim to reduce isolation.  5. Studies with no intervention.  6. Studies set in low- and middle-class income countries, skilled nursing facilities, assisted living facilities and hospitals.  7. Qualitative studies.  8. Articles not written in English. |
| Virues-Ortega (2012)^106^ | 1975 – 2009 | MEDLINE, PsycINFO and the Cochrane Library. (n = 3) | Human-pet bonding, human-animal bonding, human-animal interaction, human-pet interaction, human-dog bonding, human-dog interaction, animal ownership, animal adoption, animal therapy, animal-assisted, animal-facilitated, pet ownership, pet adoption, pet therapy, pet-assisted, pet-facilitated, dog ownership, dog adoption, dog therapy, dog-assisted, dog-facilitated, dolphin therapy, dolphin-assisted, dolphin-facilitated, equine therapy, equine-assisted, equine-facilitated, hippotherapy, therapeutic-riding, horseback-riding | Inclusion:   1. 1. Intervention studies evaluating the health-related effects of animal-assisted therapy.   Exclusion:  1. Studies which did not include animal-assisted therapy.  2. Studies that were not original research or that reported unpublished research.  3. Observational studies.  4. Studies in which the population was healthy and non-elderly. 5. Studies in which the intervention group had fewer than 5 people.  6. Studies that used anecdotal or qualitative outcome recording.  7. Studies which had no pre-test measurement.  8. Studies which reported insufficient data.  9. Secondary articles of the same study. 10. Animal assisted therapy studies that only reported isolated outcomes. |

ASSIA=Applied Social Sciences Index Abstracts; CDSR=Cochrane Database of Systematic Reviews; CENTRAL=Cochrane Central Register of Controlled Trials; CINAHL=Cumulative Index to Nursing and Allied Health Literature; DARE=Database of Abstracts of Reviews of Effects; ERIC=Education Resources Information Center; HABRI=Human-Animal Bond Research Initiative; IBSS=International Bibliography of the Social Sciences; LILACS=Latin American & Caribbean Health Sciences Literature; SCI=Science Citation Index; SIGLE=System for Information on Grey Literature in Europe; SPP=Shared Socioeconomic Pathways; SSCI=Social Science Citation Index.

**S5 Table: PRISMA Checklist**

| **Section/topic** | **#** | | **Checklist item** | | **Reported on page #** | |
| --- | --- | --- | --- | --- | --- | --- |
| **TITLE** | | | | |  | |
| Title | 1 | | Identify the report as a systematic review, meta-analysis, or both. | | 1 | |
| **ABSTRACT** | | | | |  | |
| Structured summary | 2 | | Provide a structured summary including, as applicable: background; objectives; data sources; study eligibility criteria, participants, and interventions; study appraisal and synthesis methods; results; limitations; conclusions and implications of key findings; systematic review registration number. | | 2 | |
| **INTRODUCTION** | | | | |  | |
| Rationale | 3 | | Describe the rationale for the review in the context of what is already known. | | 4 | |
| Objectives | 4 | | Provide an explicit statement of questions being addressed with reference to participants, interventions, comparisons, outcomes, and study design (PICOS). | | 4 | |
| **METHODS** | | | | |  | |
| Protocol and registration | 5 | | Indicate if a review protocol exists, if and where it can be accessed (e.g., Web address), and, if available, provide registration information including registration number. | | 6 | |
| Eligibility criteria | 6 | | Specify study characteristics (e.g., PICOS, length of follow-up) and report characteristics (e.g., years considered, language, publication status) used as criteria for eligibility, giving rationale. | | 6 | |
| Information sources | 7 | | Describe all information sources (e.g., databases with dates of coverage, contact with study authors to identify additional studies) in the search and date last searched. | | 6 | |
| Search | 8 | | Present full electronic search strategy for at least one database, including any limits used, such that it could be repeated. | | 6 | |
| Study selection | 9 | | State the process for selecting studies (i.e., screening, eligibility, included in systematic review, and, if applicable, included in the meta-analysis). | | 6-7 | |
| Data collection process | 10 | | Describe method of data extraction from reports (e.g., piloted forms, independently, in duplicate) and any processes for obtaining and confirming data from investigators. | | 8 | |
| Data items | 11 | | List and define all variables for which data were sought (e.g., PICOS, funding sources) and any assumptions and simplifications made. | | 6 | |
| Risk of bias in individual studies | 12 | | Describe methods used for assessing risk of bias of individual studies (including specification of whether this was done at the study or outcome level), and how this information is to be used in any data synthesis. | | 9 | |
| Summary measures | 13 | | State the principal summary measures (e.g., risk ratio, difference in means). | | 7 | |
| Synthesis of results | 14 | | Describe the methods of handling data and combining results of studies, if done, including measures of consistency (e.g., I^2^) for each meta-analysis. | | 8 | |
| Risk of bias across studies | | 15 | | Specify any assessment of risk of bias that may affect the cumulative evidence (e.g., publication bias, selective reporting within studies). | | n/a |
| Additional analyses | | 16 | | Describe methods of additional analyses (e.g., sensitivity or subgroup analyses, meta-regression), if done, indicating which were pre-specified. | | n/a |
| **RESULTS** | | | | | |  |
| Study selection | | 17 | | Give numbers of studies screened, assessed for eligibility, and included in the review, with reasons for exclusions at each stage, ideally with a flow diagram. | | 10/Figure 1 |
| Study characteristics | | 18 | | For each study, present characteristics for which data were extracted (e.g., study size, PICOS, follow-up period) and provide the citations. | | 10, Tables 2-4, Supplementary tables |
| Risk of bias within studies | | 19 | | Present data on risk of bias of each study and, if available, any outcome level assessment (see item 12). | | 11 |
| Results of individual studies | | 20 | | For all outcomes considered (benefits or harms), present, for each study: (a) simple summary data for each intervention group (b) effect estimates and confidence intervals, ideally with a forest plot. | | 11-15, Tables 1-4, Supplementary Table 1 and 2 |
| Synthesis of results | | 21 | | Present results of each meta-analysis done, including confidence intervals and measures of consistency. | | n/a |
| Risk of bias across studies | | 22 | | Present results of any assessment of risk of bias across studies (see Item 15). | | n/a |
| Additional analysis | | 23 | | Give results of additional analyses, if done (e.g., sensitivity or subgroup analyses, meta-regression [see Item 16]). | | n/a |
| **DISCUSSION** | | | | | |  |
| Summary of evidence | | 24 | | Summarize the main findings including the strength of evidence for each main outcome; consider their relevance to key groups (e.g., healthcare providers, users, and policy makers). | | 16 |
| Limitations | | 25 | | Discuss limitations at study and outcome level (e.g., risk of bias), and at review-level (e.g., incomplete retrieval of identified research, reporting bias). | | 18-19 |
| Conclusions | | 26 | | Provide a general interpretation of the results in the context of other evidence, and implications for future research. | | 16-18 |
| **FUNDING** | | | | | |  |
| Funding | | 27 | | Describe sources of funding for the systematic review and other support (e.g., supply of data); role of funders for the systematic review. | | See Financial Disclosure Statement |

*From:*  Moher D, Liberati A, Tetzlaff J, Altman DG, The PRISMA Group (2009). Preferred Reporting Items for Systematic Reviews and Meta-Analyses: The PRISMA Statement. PLoS Med 6(7): e1000097. doi:10.1371/journal.pmed1000097

For more information, visit: **www.prisma-statement.org**.

**Supplementary References**

1. Banks MR, Banks WA. The Effects of Animal-Assisted Therapy on Loneliness in an Elderly Population in Long-Term Care Facilities. *The Journals of Gerontology Series A: Biological Sciences and Medical Sciences*. 2002;57(7):M428-M432. doi:10.1093/gerona/57.7.M428

2. Banks MR, Banks WA. The effects of group and individual animal-assisted therapy on loneliness in residents of long-term care facilities. *Anthrozoös*. 2005;18(4):396-408. doi:10.2752/089279305785593983

3. Riddick CC. Health, Aquariums, and the Non-Institutionalized Elderly. *Marriage & Family Review*. 1985;8(3-4):163-173. doi:10.1300/J002v08n03_12

4. Sollami A, Gianferrari E, Alfieri M, Artioli G, Taffurelli C. Pet therapy: an effective strategy to care for the elderly? An experimental study in a nursing home. *Acta Biomed*. 2017;88(1S):25-31. doi:10.23750/abm.v88i1 -S.6281

5. MacIntyre I, Corradetti P, Roberts J, Browne G, Watt S, Lane A. Pilot study of a visitor volunteer programme for community elderly people receiving home health care. *Health & Social Care in the Community*. 1999;7(3):225-232. doi:10.1046/j.1365-2524.1999.00178.x

6. Mulligan MA, Bennett R. Assessment of Mental Health and Social Problems during Multiple Friendly Visits: The Development and Evaluation of a Friendly Visiting Program for the Isolated Elderly. *Int J Aging Hum Dev*. 1978;8(1):43-65. doi:10.2190/6F9D-2FT9-JFQB-M8R2

7. Rook KS, Sorkin DH. Fostering social ties through a volunteer role: Implications for older-adults’ psychological health. *International Journal of Aging & Human Development*. 2003;57(4):313-337. doi:10.2190/NBBN-EU3H-4Q1N-UXHR

8. Alaviani M, Khosravan S, Alami A, Moshki M. The Effect of a Multi-Strategy Program on Developing Social Behaviors Based on Pender’s Health Promotion Model to Prevent Loneliness of Old Women Referred to Gonabad Urban Health Centers. *Int J Community Based Nurs Midwifery*. 2015;3(2):132-140.

9. Honigh - de Vlaming R. Healthy Ageing: prevention of loneliness among elderly people : evaluation of a complex intervention in public health practice. Published online 2013. https://edepot.wur.nl/252853

10. Lupton BS, Fonnebo V, Sogaard AJ, Fylkesnes K. The Finnmark Intervention Study: do community-based intervention programmes threaten self-rated health and well-being? Experiences from Batsfjord, a fishing village in North Norway. *European Journal of Public Health*. 2005;15(1):91-96. doi:10.1093/eurpub/cki101

11. de Craen AJM, Gussekloo J, Blauw GJ, Willems CG, Westendorp RGJ. Randomised controlled trial of unsolicited occupational therapy in community-dwelling elderly people: The LOTIS trial. *Plos Clinical Trials*. 2006;1(1):e2. doi:10.1371/journal.pctr.0010002

12. Melis RJF, van Eijken MIJ, Teerenstra S, et al. A randomized study of a multidisciplinary program to intervene on geriatric syndromes in vulnerable older people who live at home (Dutch EASYcare Study). *J Gerontol A Biol Sci Med Sci*. 2008;63(3):283-290. doi:10.1093/gerona/63.3.283

13. Clarke M, Clarke SJ, Jagger C. Social Intervention and the Elderly:A Randomized Controlled Trial. *American Journal of Epidemiology*. 1992;136(12):1517-1523. doi:10.1093/oxfordjournals.aje.a116473

14. McEwan RT, Davison N, Forster DP, Pearson P, Stirling E. Screening elderly people in primary care: a randomized controlled trial. *Br J Gen Pract*. 1990;40(332):94-97.

15. Morrow-Howell N, Becker-Kemppainen S, Judy L. Evaluating an Intervention for the Elderly at Increased Risk of Suicide. *Research on Social Work Practice*. 1998;8(1):28-46. doi:10.1177/104973159800800104

16. Ollonqvist K, Palkeinen H, Aaltonen T, et al. Alleviating Loneliness among Frail Older People – Findings from a Randomised Controlled Trial. *International Journal of Mental Health Promotion*. 2008;10(2):26-34. doi:10.1080/14623730.2008.9721760

17. Parsons JGM, Sheridan N, Rouse P, Robinson E, Connolly M. A Randomized Controlled Trial to Determine the Effect of a Model of Restorative Home Care on Physical Function and Social Support Among Older People. *Archives of Physical Medicine and Rehabilitation*. 2013;94(6):1015-1022. doi:10.1016/j.apmr.2013.02.003

18. Tse MMY, Tang SK, Wan VTC, Vong SKS. The Effectiveness of Physical Exercise Training in Pain, Mobility, and Psychological Well-being of Older Persons Living in Nursing Homes. *Pain Management Nursing*. 2014;15(4):778-788. doi:10.1016/j.pmn.2013.08.003

19. Cohen GD, Perlstein S, Chapline J, Kelly J, Firth KM, Simmens S. The Impact of Professionally Conducted Cultural Programs on the Physical Health, Mental Health, and Social Functioning of Older Adults. *The Gerontologist*. 2006;46(6):726-734. doi:10.1093/geront/46.6.726

20. Yap AF, Kwan YH, Tan CS, Ibrahim S, Bin Ang S. Rhythm-centred music making in community living elderly: a randomized pilot study. *Bmc Complementary and Alternative Medicine*. 2017;17:311. doi:10.1186/s12906-017-1825-x

21. Pynnonen K, Tormakangas T, Rantanen T, Tiikkainen P, Kallinen M. Effect of a social intervention of choice vs. control on depressive symptoms, melancholy, feeling of loneliness, and perceived togetherness in older Finnish people: a randomized controlled trial. *Aging & Mental Health*. 2018;22(1):77-84. doi:10.1080/13607863.2016.1232367

22. Routasalo PE, Tilvis RS, Kautiainen H, Pitkala KH. Effects of psychosocial group rehabilitation on social functioning, loneliness and well-being of lonely, older people: randomized controlled trial. *Journal of Advanced Nursing*. 2009;65(2):297-305. doi:10.1111/j.1365-2648.2008.04837.x

23. Boen H, Dalgard OS, Johansen R, Nord E. A randomized controlled trial of a senior centre group programme for increasing social support and preventing depression in elderly people living at home in Norway. *Bmc Geriatrics*. 2012;12:20. doi:10.1186/1471-2318-12-20

24. Banks MR, Willoughby LM, Banks WA. Animal-Assisted Therapy and Loneliness in Nursing Homes: Use of Robotic versus Living Dogs. *Journal of the American Medical Directors Association*. 2008;9(3):173-177. doi:10.1016/j.jamda.2007.11.007

25. Jessen J, Cardiello F, Baun MM. Avian Companionship in Alleviation of Depression, Loneliness, and Low Morale of Older Adults in Skilled Rehabilitation Units. *Psychol Rep*. 1996;78(1):339-348. doi:10.2466/pr0.1996.78.1.339

26. Robinson H, MacDonald B, Kerse N, Broadbent E. The Psychosocial Effects of a Companion Robot: A Randomized Controlled Trial. *Journal of the American Medical Directors Association*. 2013;14(9):661-667. doi:10.1016/j.jamda.2013.02.007

27. Heller K, Thompson MG, Trueba PE, Hogg JR, Vlachos-Weber I. Peer support telephone dyads for elderly women: Was this the wrong intervention? *American Journal of Community Psychology*. 1991;19(1):53-74. doi:10.1007/BF00942253

28. Mountain GA, Hind D, Gossage-Worrall R, et al. “Putting Life in Years” (PLINY) telephone friendship groups research study: pilot randomised controlled trial. *Trials*. 2014;15:141. doi:10.1186/1745-6215-15-141

29. Hind D, Mountain G, Gossage-Worrall R, et al. Putting Life in Years (PLINY): a randomised controlled trial and mixed-methods process evaluation of a telephone friendship intervention to improve mental well-being in independently living older people. *Public Health Research*. 2014;2(7):1-222. doi:10.3310/phr02070

30. Schulz R. Effects of control and predictability on the physical and psychological well-being of the institutionalized aged. *J Pers Soc Psychol*. 1976;33(5):563-573. doi:10.1037//0022-3514.33.5.563

31. Bouwman TE, Aartsen MJ, van Tilburg TG, Stevens NL. Does stimulating various coping strategies alleviate loneliness? Results from an online friendship enrichment program. *Journal of Social and Personal Relationships*. 2017;34(6):793-811. doi:10.1177/0265407516659158

32. Martina CMS, Stevens NL. Breaking the cycle of loneliness? Psychological effects of a friendship enrichment program for older women. *Aging & Mental Health*. 2006;10(5):467-475. doi:10.1080/13607860600637893

33. Stevens NL, Martina CMS, Westerhof GJ. Meeting the need to belong: Predicting effects of a friendship enrichment program for older women. *Gerontologist*. 2006;46(4):495-502. doi:10.1093/geront/46.4.495

34. Tilburg NS Theo Van. Stimulating Friendship in Later Life: A Strategy for Reducing Loneliness Among Older Women. *Educational Gerontology*. 2000;26(1):15-35. doi:10.1080/036012700267376

35. Cohen-Mansfield J, Hazan H, Lerman Y, Shalom V, Birkenfeld S, Cohen R. Efficacy of the I-SOCIAL intervention for loneliness in old age: Lessons from a randomized controlled trial. *Journal of Psychiatric Research*. 2018;99:69-75. doi:10.1016/j.jpsychires.2018.01.014

36. Kremers IP, Steverink N, Albersnagel FA, Slaets JPJ. Improved self-management ability and well-being in older women after a short group intervention. *Aging & Mental Health*. 2006;10(5):476-484. doi:10.1080/13607860600841206

37. Mountain G, Windle G, Hind D, et al. A preventative lifestyle intervention for older adults (lifestyle matters): a randomised controlled trial. *Age and Ageing*. 2017;46(4):627-634. doi:10.1093/ageing/afx021

38. Ruffing-Rahal MA. Evaluation of group health promotion with community-dwelling older women. *Public Health Nursing*. 1994;11(1):38-48. doi:10.1111/j.1525-1446.1994.tb00388.x

39. Seepersad SS. Understanding and helping the lonely: An evaluation of the Luv Program. *(Unpublished doctoral dissertation)*. Published online 2005. University of Illinois at Urbana-Champaign.

40. Hall N, Beck PD, Johnson D, Mackinnon K, Gutman G, Glick N. Randomized Trial of a Health Promotion Program For Frail Elders. *Can J Aging*. 1992;11(1):72-91. doi:10.1017/S0714980800014537

41. van Rossum E, Frederiks CM, Philipsen H, Portengen K, Wiskerke J, Knipschild P. Effects of preventive home visits to elderly people. *BMJ*. 1993;307(6895):27-32. doi:10.1136/bmj.307.6895.27

42. Dickens AP, Richards SH, Hawton A, et al. An evaluation of the effectiveness of a community mentoring service for socially isolated older people: a controlled trial. *Bmc Public Health*. 2011;11:218. doi:10.1186/1471-2458-11-218

43. Shapira N, Barak A, Gal I. Promoting older adults’ well-being through Internet training and use. *Aging & Mental Health*. 2007;11(5):477-484. doi:10.1080/13607860601086546

44. Slegers K, van Boxtel MPJ, Jolles J. Effects of computer training and Internet usage on the well-being and quality of life of older adults: a randomized, controlled study. *J Gerontol B Psychol Sci Soc Sci*. 2008;63(3):P176-184. doi:10.1093/geronb/63.3.p176

45. White H, McConnell E, Clipp E, et al. Surfing the Net in Later Life: A Review of the Literature and Pilot Study of Computer Use and Quality of Life. *J Appl Gerontol*. 1999;18(3):358-378. doi:10.1177/073346489901800306

46. White H, McConnell E, Clipp E, et al. A randomized controlled trial of the psychosocial impact of providing internet training and access to older adults. *Aging & Mental Health*. 2002;6(3):213-221. doi:10.1080/13607860220142422

47. Woodward AT, Freddolino PP, Blaschke-Thompson CM, et al. Technology and Aging Project: Training Outcomes and Efficacy from a Randomized Field Trial. *Ageing Int*. 2011;36(1):46-65. doi:10.1007/s12126-010-9074-z

48. Bickmore TW, Caruso L, Clough-Gorr K, Heeren T. ‘It’s just like you talk to a friend’ relational agents for older adults. *Interacting with Computers*. 2005;17(6):711-735. doi:10.1016/j.intcom.2005.09.002

49. Dowd AJ, Schmader T, Sylvester BD, et al. Effects of Social Belonging and Task Framing on Exercise Cognitions and Behavior. *Journal of Sport and Exercise Psychology*. 2014;36(1):80-92. doi:10.1123/jsep.2013-0114

50. Kamegaya T, Araki Y, Kigure H, Long-Term-Care Prevention Team of Maebashi City, Yamaguchi H. Twelve-week physical and leisure activity programme improved cognitive function in community-dwelling elderly subjects: a randomized controlled trial: Preventing cognitive decline. *Psychogeriatrics*. 2014;14(1):47-54. doi:10.1111/psyg.12038

51. Iliffe S, Kendrick D, Morris R, et al. Multicentre cluster randomised trial comparing a community group exercise programme and home-based exercise with usual care for people aged 65 years and over in primary care. *Health Technology Assessment*. 2014;18(49):1-106. doi:10.3310/hta18490

52. Maki Y, Ura C, Yamaguchi T, et al. Effects of Intervention Using a Community-Based Walking Program for Prevention of Mental Decline: A Randomized Controlled Trial. *J Am Geriatr Soc*. 2012;60(3):505-510. doi:10.1111/j.1532-5415.2011.03838.x

53. McAuley E, Blissmer S, Marquez DX, Jerome GJ, Kramer AF, Katula J. Social relations, physical activity, and well-being in older adults. *Preventive Medicine*. 2000;31(5):608-617. doi:10.1006/pmed.2000.0740

54. Mutrie N, Doolin O, Fitzsimons CF, et al. Increasing older adults’ walking through primary care: results of a pilot randomized controlled trial. *Family Practice*. 2012;29(6):633-642. doi:10.1093/fampra/cms038

55. Tarazona-Santabalbina FJ, Gómez-Cabrera MC, Pérez-Ros P, et al. A Multicomponent Exercise Intervention that Reverses Frailty and Improves Cognition, Emotion, and Social Networking in the Community-Dwelling Frail Elderly: A Randomized Clinical Trial. *Journal of the American Medical Directors Association*. 2016;17(5):426-433. doi:10.1016/j.jamda.2016.01.019

56. Perkins P. Impact of a Horticultural Therapy Program on the Well-Being of Low-Income Community Dwelling Older Adults. *Acta Hortic*. 2012;(954):123-132. doi:10.17660/ActaHortic.2012.954.14

57. Tse MMY. Therapeutic effects of an indoor gardening programme for older people living in nursing homes. *Journal of Clinical Nursing*. 2010;19(7-8):949-958. doi:10.1111/j.1365-2702.2009.02803.x

58. Bell CS, Fain E, Daub J, et al. Effects of Nintendo Wii on Quality of Life, Social Relationships, and Confidence to Prevent Falls. *Physical & Occupational Therapy In Geriatrics*. 2011;29(3):213-221. doi:10.3109/02703181.2011.559307

59. Kahlbaugh PE, Sperandio AJ, Carlson AL, Hauselt J. Effects of Playing Wii on Well-Being in the Elderly: Physical Activity, Loneliness, and Mood. *Activities, Adaptation & Aging*. 2011;35(4):331-344. doi:10.1080/01924788.2011.625218

60. Jung Y, Li KJ, Janissa NS, Gladys WLC, Lee KM. Games for a better life: effects of playing Wii games on the well-being of seniors in a long-term care facility. In: *Proceedings of the Sixth Australasian Conference on Interactive Entertainment - IE ’09*. ACM Press; 2009:1-6. doi:10.1145/1746050.1746055

61. Xu X, Li J, Pham TP, Salmon CT, Theng Y-L. Improving Psychosocial Well-Being of Older Adults Through Exergaming: The Moderation Effects of Intergenerational Communication and Age Cohorts. *Games for Health Journal*. 2016;5(6):389-397. doi:10.1089/g4h.2016.0060

62. Winstead V, Yost EA, Cotten SR, Berkowsky RW, Anderson WA. The Impact of Activity Interventions on the Well-Being of Older Adults in Continuing Care Communities. *J Appl Gerontol*. 2014;33(7):888-911. doi:10.1177/0733464814537701

63. Cobb NK, Poirier J. Effectiveness of a Multimodal Online Well-Being Intervention. *American Journal of Preventive Medicine*. 2014;46(1):41-48. doi:10.1016/j.amepre.2013.08.018

64. Conoley C, Garber R. Effects of Reframing and Self-Control Directives on Loneliness, Depression, and Controllability. *Journal of Counseling Psychology*. 1985;32(1):139-142.

65. Dodge HH, Zhu J, Mattek NC, et al. Web-enabled conversational interactions as a method to improve cognitive functions: Results of a 6-week randomized controlled trial. *Alzheimer’s & Dementia: Translational Research & Clinical Interventions*. 2015;1(1):1-12. doi:10.1016/j.trci.2015.01.001

66. McWhirter BT, Horan JJ. Construct validity of cognitive-behavioral treatments for intimate and social loneliness. *Current Psychology*. 1996;15(1):42-52. doi:10.1007/BF02686933

67. Winningham RG, Pike NL. A cognitive intervention to enhance institutionalized older adults’ social support networks and decrease loneliness. *Aging & Mental Health*. 2007;11(6):716-721. doi:10.1080/13607860701366228

68. Kuru Alici N, Bahceli PZ, Emiroglu ON. The preliminary effects of laughter therapy on loneliness and death anxiety among older adults living in nursing homes: A nonrandomised pilot study. *International Journal of Older People Nursing*. 2018;13(4):e12206. doi:10.1111/opn.12206

69. Elsherbiny MMK, Al Maamari RH. The Effectiveness of Logotherapy in Mitigating the Social Isolation of neglected Institutionalised Older People. *British Journal of Social Work*. 2018;48(4):1090-1108. doi:10.1093/bjsw/bcy043

70. Adair KC, Fredrickson BL, Castro-Schilo L, Kim S, Sidberry S. Present with You: Does Cultivated Mindfulness Predict Greater Social Connection Through Gains in Decentering and Reductions in Negative Emotions? *Mindfulness*. 2018;9(3):737-749. doi:10.1007/s12671-017-0811-1

71. Creswell JD, Irwin MR, Burklund LJ, et al. Mindfulness-Based Stress Reduction training reduces loneliness and pro-inflammatory gene expression in older adults: A small randomized controlled trial. *Brain Behavior and Immunity*. 2012;26(7):1095-1101. doi:10.1016/j.bbi.2012.07.006

72. Zhang N, Fan F, Huang S, Rodriguez MA. Mindfulness training for loneliness among Chinese college students: A pilot randomized controlled trial. *International Journal of Psychology*. 2018;53(5):373-378. doi:10.1002/ijop.12394

73. Chiang K-J, Chu H, Chang H-J, et al. The effects of reminiscence therapy on psychological well-being, depression, and loneliness among the institutionalized aged. *International Journal of Geriatric Psychiatry*. 2010;25(4):380-388. doi:10.1002/gps.2350

74. Westerhof GJ, Korte J, Eshuis S, Bohlmeijer ET. Precious memories: a randomized controlled trial on the effects of an autobiographical memory intervention delivered by trained volunteers in residential care homes. *Aging & Mental Health*. 2018;22(11):1494-1501. doi:10.1080/13607863.2017.1376311

75. Chan AWK, Yu DSF, Choi KC. Effects of tai chi qigong on psychosocial well-being among hidden elderly, using elderly neighborhood volunteer approach: a pilot randomized controlled trial. *Clinical Interventions in Aging*. 2017;12:85-96. doi:10.2147/CIA.S124604

76. Wikström B-M. Social interaction associated with visual art discussions: A controlled intervention study. *Aging & Mental Health*. 2002;6(1):82-87. doi:10.1080/13607860120101068

77. Andersson L. Intervention against loneliness in a group of elderly women: An impact evaluation. *Social Science & Medicine*. 1985;20(4):355-364. doi:10.1016/0277-9536(85)90010-3

78. Baumgarten M, Thomas D, Poulin de Courval L, Infante-Rivard C. Evaluation of a mutual help network for the elderly residents of planned housing. *Psychol Aging*. 1988;3(4):393-398. doi:10.1037//0882-7974.3.4.393

79. Czaja SJ, Boot WR, Charness N, Rogers WA, Sharit J. Improving Social Support for Older Adults Through Technology: Findings From the PRISM Randomized Controlled Trial. *The Gerontologist*. 2018;58(3):467-477. doi:10.1093/geront/gnw249

80. Lökk J. Emotional and Social Effects of a Controlled Intervention Study in a Day-Care Unit for Elderly Patients. *Scandinavian Journal of Primary Health Care*. 1990;8(3):165-172. doi:10.3109/02813439008994951

81. Saito T, Kai I, Takizawa A. Effects of a program to prevent social isolation on loneliness, depression, and subjective well-being of older adults: A randomized trial among older migrants in Japan. *Archives of Gerontology and Geriatrics*. 2012;55(3):539-547. doi:10.1016/j.archger.2012.04.002

82. Tsai H-H, Tsai Y-F, Wang H-H, Chang Y-C, Chu HH. Videoconference program enhances social support, loneliness, and depressive status of elderly nursing home residents. *Aging & Mental Health*. 2010;14(8):947-954. doi:10.1080/13607863.2010.501057

83. Tsai H-H, Tsai Y-F. Changes in Depressive Symptoms, Social Support, and Loneliness Over 1 Year After a Minimum 3-Month Videoconference Program for Older Nursing Home Residents. *Journal of Medical Internet Research*. 2011;13(4):e93. doi:10.2196/jmir.1678

84. Abbott R, Orr N, McGill P, et al. How do “robopets” impact the health and well-being of residents in care homes? A systematic review of qualitative and quantitative evidence. *International Journal of Older People Nursing*. 2019;14(3):e12239. doi:10.1111/opn.12239

85. Baker S, Warburton J, Waycott J, et al. Combatting social isolation and increasing social participation of older adults through the use of technology: A systematic review of existing evidence. *Australas J Ageing*. 2018;37(3):184-193. doi:10.1111/ajag.12572

86. Bessaha ML, Sabbath EL, Morris Z, Malik S, Scheinfeld L, Saragossi J. A Systematic Review of Loneliness Interventions Among Non-elderly Adults. *Clinical Social Work Journal*. 2020;48(1):110-125. doi:10.1007/s10615-019-00724-0

87. Cattan M, White M, Bond J, Learmouth A. Preventing social isolation and loneliness among older people: a systematic review of health promotion interventions. *Ageing and Society*. 2005;25(01):41-67. doi:10.1017/S0144686X04002594

88. Cohen-Mansfield J, Perach R. Interventions for Alleviating Loneliness Among Older Persons: A Critical Review. *American Journal of Health Promotion*. 2015;29(3):E109-E125. doi:10.4278/ajhp.130418-LIT-182

89. Coll-Planas L, Nyqvist F, Puig T, Urrutia G, Sola I, Monteserin R. Social capital interventions targeting older people and their impact on health: a systematic review. *Journal of Epidemiology and Community Health*. 2017;71(7):663-672. doi:10.1136/jech-2016-208131

90. Dickens AP, Richards SH, Greaves CJ, Campbell JL. Interventions targeting social isolation in older people: a systematic review. *Bmc Public Health*. 2011;11:647. doi:10.1186/1471-2458-11-647

91. Forsman AK, Nordmyr J, Matosevic T, Park A-L, Wahlbeck K, McDaid D. Promoting mental wellbeing among older people: technology-based interventions. *Health Promotion International*. 2018;33(6):1042-1054. doi:10.1093/heapro/dax047

92. Gee NR, Mueller MK. A Systematic Review of Research on Pet Ownership and Animal Interactions among Older Adults. *Anthrozoös*. 2019;32(2):183-207. doi:10.1080/08927936.2019.1569903

93. Hagan R, Manktelow R, Taylor BJ, Mallett J. Reducing loneliness amongst older people: a systematic search and narrative review. *Aging & Mental Health*. 2014;18(6):683-693. doi:10.1080/13607863.2013.875122

94. Kall A, Shafran R, Lindegaard T, et al. A Common Elements Approach to the Development of a Modular Cognitive Behavioral Theory for Chronic Loneliness. *Journal of Consulting and Clinical Psychology*. 2020;88(3):269-282. doi:10.1037/ccp0000454

95. Li J, Erdt M, Chen L, Cao Y, Lee S-Q, Theng Y-L. The Social Effects of Exergames on Older Adults: Systematic Review and Metric Analysis. *Journal of Medical Internet Research*. 2018;20(6):e10486. doi:10.2196/10486

96. Masi CM, Chen H-Y, Hawkley LC, Cacioppo JT. A Meta-Analysis of Interventions to Reduce Loneliness. *Personality and Social Psychology Review*. 2011;15(3):219-266. doi:10.1177/1088868310377394

97. Mikkelsen ASB, Petersen S, Dragsted AC, Kristiansen M. Social Interventions Targeting Social Relations Among Older People at Nursing Homes: A Qualitative Synthesized Systematic Review. *Inquiry-the Journal of Health Care Organization Provision and Financing*. 2019;56:0046958018823929. doi:10.1177/0046958018823929

98. Nnabuko U, Anderson S. The effect of ICT on social support in healthcare: a systematic review. *IJCSIS*. 2018;13(1):14-32. doi:10.33965/ijcsis_2018130102

99. Pels F, Kleinert J. Loneliness and physical activity: A systematic review. *International Review of Sport and Exercise Psychology*. 2016;9(1):231-260. doi:10.1080/1750984X.2016.1177849

100. Poscia A, Stojanovic J, La Milia DI, et al. Interventions targeting loneliness and social isolation among the older people: An update systematic review. *Experimental Gerontology*. 2018;102:133-144. doi:10.1016/j.exger.2017.11.017

101. Quan NG, Lohman MC, Resciniti NV, Friedman DB. A systematic review of interventions for loneliness among older adults living in long-term care facilities. *Aging & Mental Health*. Published online October 11, 2019:1-11. doi:10.1080/13607863.2019.1673311

102. Shvedko A, Whittaker AC, Thompson JL, Greig CA. Physical activity interventions for treatment of social isolation, loneliness or low social support in older adults: A systematic review and meta-analysis of randomised controlled trials. *Psychology of Sport and Exercise*. 2018;34:128-137. doi:10.1016/j.psychsport.2017.10.003

103. Siette J, Cassidy M, Priebe S. Effectiveness of befriending interventions: a systematic review and meta-analysis. *BMJ Open*. 2017;7(4):e014304. doi:10.1136/bmjopen-2016-014304

104. Sims-Gould J, Tong CE, Wallis-Mayer L, Ashe MC. Reablement, Reactivation, Rehabilitation and Restorative Interventions With Older Adults in Receipt of Home Care: A Systematic Review. *Journal of the American Medical Directors Association*. 2017;18(8):653-663. doi:10.1016/j.jamda.2016.12.070

105. Veazie S, Gilbert J, Winchell K, Paynter R, Guise J-M. *Addressing Social Isolation To Improve the Health of Older Adults: A Rapid Review*. Agency for Healthcare Research and Quality (AHRQ); 2019. Accessed July 7, 2020. https://effectivehealthcare.ahrq.gov/topics/social-isolation/rapid-product

106. Virues-Ortega J, Pastor-Barriuso R, Castellote JM, Poblacion A, de Pedro-Cuesta J. Effect of animal-assisted therapy on the psychological and functional status of elderly populations and patients with psychiatric disorders: a meta-analysis. *Health Psychology Review*. 2012;6(2):197-221. doi:10.1080/17437199.2010.534965
