# Supplementary material for: Interventions to reduce social isolation and loneliness during COVID-19 physical distancing measures: A rapid systematic review
Source: PLoS One. 2021 Feb 17;16(2):e0247139. doi: 10.1371/journal.pone.0247139 (PMC7888614; doi:10.1371/journal.pone.0247139)
Supplement: Attachment — Submitted filename: Response to reviewers.docx [file pone.0247139.s002.docx]

**Response to reviewers**

We would like to thank the academic editor and reviewer for their helpful comments. Our detailed point-by-point responses are provided below.

Editor:

This is an interesting timely and well conducted review.

*Many thanks for your kind comments.*

The abstract is well written and comprehensive.

Introduction: it might be useful to update this to the current situation with regard to COVID-19 in the UK and internationally particularly around restrictions being reimposed.

*We agree. We have updated lines 75-77 (of the tracked-changes manuscript) to reflect the re-introduction of national lockdowns.*

Methods: Well described and seems robust. The categories of loneliness reported are justified. Exclusions are justified. One limitation is that more recent studies - those not included in a review - will not be captured by the methodology. This decision (to do a rapid review) should be more clearly justified In the method. Narrative synthesis is justified.

*We agree. We have clarified the need to conduct a rapid review to urgently inform healthcare policy decisions in lines 105-106. Furthermore, we have acknowledged as a limitation in the* Discussion *that some recent studies may not have been captured and explained how we sought to mitigate this.*

Results: Would it also be worth thinking about interventions that would be feasible under strict covert measures, but not quite shielding, which seems to be the current situation in the UK at the moment? I think this would just require another section/ paragraph in the results.

*Within the 58 studies, we have already included all the interventions which do not entirely meet the shielding measures, but would be feasible with modification (these were classed as “*Potentially Feasible”*, and required modification to bring them in alignment with the principles of shielding). We feel that with the ongoing changes to government COVID-19 restrictions, a standardised approach to classify interventions using the original definition of shielding is most appropriate. Included interventions will therefore be feasible regardless of the current severity of restrictions and will remain feasible as these restrictions change in the future.*

It would be good to know which categories of Gardiner's framework the non-viable and unclear interventions were in.

*We have expanded the paragraph on “*Not feasible” *or only “*Part feasible” *interventions to also detail the types of interventions that were excluded. Full details of these interventions can be found in Supplementary Table 1.*

It would have been good to look at the effectiveness of the programmes on different populations; for example, older people who may be particularly vulnerable and at risk during COVID-19 restrictions - there is an oblique reference to this in the discussion (second paragraph on page 16) but it should be made more explicit that older adults are a vulnerable category.

*We are grateful for this suggestion. We have added a subsection to the* Results *that covers effective interventions for vulnerable groups. Furthermore, in the* Discussion *we have discussed the current provision for interventions targeting certain vulnerable groups.*

Discussion: Rather than interventions that target the whole age population, would it not be better to think about future interventions that are targeted at specific age groups?

*We agree. We have re-structured the* Discussion *to consider different age groups.*

Reviewer:

Many thanks to the authors for this submission. This research offers a timely and original contribution to the rapidly emerging Covid-19 field of research.

The rationale and methodological approaches are transparent and have been expertly managed to produce the review.

The findings and implications of the rapid review are of relevance across a number of professional disciplines as well as for policy and practice in helping to inform responses which offer evidence based interventions to reduce social isolation at this time.

*Many thanks for your kind comments.*

Two additional aspects to the review could be considered. One is touched upon on line 357. 1) Addressing inequality in access/inclusion, inclusive of digital literacy could be more fully considered. A couple of ways this would be addressed is by highlighting if any of the reviewed studies provide a blueprint/demonstrate good practice in the area of accessibility for groups at risk of inequality in access such as older age adults - many of whom are facing the highest levels of social isolation (previously shielding). Some references to those populations who are facing highest levels of social isolation and loneliness would be valuable to evidence, so thought can be given to the design/tailoring to the populations affected. At the moment, the review is perhaps quite homogeneous in how it considers social distancing/covid related measures on 'people'. However as said previously, we know that this pandemic is reinforcing inequalities within specific populations such as those on low income, older age adults, BME groups (cultural competency and representations of mental health may be relevant to mention). It is possible to pull out a bit more about the participant/population characteristics from the review?

*We agree. We have added sections to the* Results *and* Discussion *which discusses the availability of interventions for vulnerable groups and highlighted the lack of effective interventions for some of these groups. We were unable to find studies providing a blueprint for increasing the accessibility of interventions, however we have warned of the necessity to consider accessibility when seeking to deliver interventions in the* Discussion*. Likewise, we have discussed the different types of accessibility (educational, economic and infrastructure) that may be required to facilitate intervention delivery.*

*Full details of the participant characteristics for each study have been extracted and can be found in the Supplementary Tables. We have clarified the reference to this in line 231.*

Furthermore, 2) it may be valuable to align some of the findings to existing social support which has been offered through Government_ NHS provision during Covid-19 period thus far. A quick check indicates that these are broadly psychoeducational, befriending in local community and psychological referral. It would be valuable to make a link in discussion which situates the findings of the review within a comment to current provision and how this provision can be improved, re-orientated to take into account the review findings about efficacy of interventions.

*We agree. We have added a section to the* Discussion *detailing the current efforts and services for loneliness and social isolation and summarising how this review adds to this current provision. We have further highlighted the need for collaboration to improve loneliness and isolation and address the growing inequalities that may be worsened by unequal access to the currently available interventions.*

Many thanks again for submitting this research article.
